# Eros is a novel transmembrane protein that controls the phagocyte respiratory burst and is essential for innate immunity

**DOI:** 10.1084/jem.20161382

**Published:** 2017-04-03

**Authors:** David C. Thomas, Simon Clare, John M. Sowerby, Mercedes Pardo, Jatinder K. Juss, David A. Goulding, Louise van der Weyden, Daniel Storisteanu, Ananth Prakash, Marion Espéli, Shaun Flint, James C. Lee, Kim Hoenderdos, Leanne Kane, Katherine Harcourt, Subhankar Mukhopadhyay, Yagnesh Umrania, Robin Antrobus, James A. Nathan, David J. Adams, Alex Bateman, Jyoti S. Choudhary, Paul A. Lyons, Alison M. Condliffe, Edwin R. Chilvers, Gordon Dougan, Kenneth G.C. Smith

**Affiliations:** 1 Department of Medicine, University of Cambridge, University of Cambridge School of Clinical Medicine, Cambridge CB2 0QQ, England, UK; 2 Cambridge Institute for Medical Research, University of Cambridge School of Clinical Medicine, Wellcome Trust/MRC Building, Cambridge Biomedical Campus, Cambridge CB2 0XY, England, UK; 3 Wellcome Trust Sanger Institute, Wellcome Trust Genome Campus, Hinxton, Cambridgeshire CB10 1SA, England, UK; 4 European Molecular Biology Laboratory, European Bioinformatics Institute, Wellcome Trust Genome Campus, Hinxton, Cambridge CB10 1SD, England, UK

## Abstract

The phagocyte respiratory burst is crucial for innate immunity. The transfer of electrons to oxygen is mediated by a membrane-bound heterodimer, comprising gp91*phox* and p22*phox* subunits. Deficiency of either subunit leads to severe immunodeficiency. We describe Eros (essential for reactive oxygen species), a protein encoded by the previously undefined mouse gene *bc017643*, and show that it is essential for host defense via the phagocyte NAPDH oxidase. Eros is required for expression of the NADPH oxidase components, gp91*phox* and p22*phox*. Consequently, *Eros*-deficient mice quickly succumb to infection. *Eros* also contributes to the formation of neutrophil extracellular traps (NETS) and impacts on the immune response to melanoma metastases. *Eros* is an ortholog of the plant protein Ycf4, which is necessary for expression of proteins of the photosynthetic photosystem 1 complex, itself also an NADPH oxio-reductase. We thus describe the key role of the previously uncharacterized protein Eros in host defense.

## Abstract (precis)

Thomas et al. show that a novel protein, Eros, controls the abundance of components of the phagocyte NADPH oxidase, making it essential for the phagocyte respiratory burst and defense against common infections.

## Introduction

Infectious diseases cause major morbidity and mortality worldwide. For example, specific serovars of *Salmonella* such as typhi and paratyphi cause typhoid fever (enteric fever), whereas nontyphi *Salmonella* can also cause invasive systemic infection (Dougan et al., 2011; Gilchrist et al., 2015). Both can be fatal, particularly in immunocompromised hosts. Our understanding of the immune response to such common and serious pathogens remains incomplete, though infection of mice with *S.* Typhimurium provides a useful model of immunity to this bacterium and others that colonize macrophages (Chatfield et al., 1992; Conlan, 1997; Richter-Dahlfors et al., 1997; Mastroeni et al., 2000; Vazquez-Torres et al., 2000a, 2004; Burton et al., 2014). Specific elements of both innate and adaptive immunity are needed to control *Salmonella* and other intracellular pathogens. Mononuclear phagocytes, particularly those in the liver and spleen, restrict bacterial replication in the initial phase of a primary invasive infection. Control of the pathogen requires (i) the effective initiation of an inflammatory response via TLR (Vazquez-Torres et al., 2004) and NOD-like receptors (NLRs; Broz et al., 2012), (ii) the production of inflammatory cytokines, such as IFN-γ and TNF (Nakano et al., 1990), and (iii) the activation of an arsenal of antibacterial strategies within the phagosome, including the generation of the phagocyte respiratory burst, the generation of reactive nitrogen species (Burton et al., 2014) and the use of efflux pumps to deprive the bacteria of divalent cations (Vidal et al., 1995; Mastroeni et al., 2000; Vazquez-Torres et al., 2000a).

The importance of these strategies is well documented, but the phagocyte respiratory burst is of particular interest because it has a nonredundant role in preventing overwhelming infection that is highly conserved between mouse (Mastroeni et al., 2000; Vazquez-Torres et al., 2000a,b) and man (van den Berg et al., 2009). The respiratory burst is a process by which superoxide and hydrogen peroxide ions are generated within the phagosome and at the cell surface. The ions are likely to be both directly and indirectly cytotoxic to the bacteria (and fungi), as the production of superoxide and hydrogen peroxide anions also activates other anti-bacterial mechanisms (Segal, 2005). Hydrogen peroxide reacts with halide ions (such as chloride or iodide) to produce the anti-bacterial compound, hypochlorite (Klebanoff et al., 1966; Klebanoff, 1967). Crucially, this process is catalyzed by myeloperoxidase, which is abundant in neutrophil granules. Hypochlorite, the product of this reaction, is itself a potent microbiocidal agent. It is also a precursor of the chloramines, which are oxidized halogens that also exhibit antimicrobial activity and are formed by the reaction of oxidized halogens with ammonia or amines (Babior, 1984).

The reactive oxygen species are generated by the phagocyte NADPH oxidase, a multiprotein complex that comprises both membrane bound and cytosolic components. The membrane-associated heterodimer, cytochrome b558, consists of gp91*phox* and p22*phox* subunits (Segal, 1987; Teahan et al., 1987) and is essential for electron transfer from NADPH to molecular oxygen. The cytosolic components p47*phox* (Kim and Dinauer, 2006), p67*phox*, p40*phox*, and the small G proteins Rac1 and Rac2 (Roberts et al., 1999; Yamauchi et al., 2004) are required for full activation. Deficiencies in individual subunits of the complex cause chronic granulomatous disease (CGD; Berendes et al., 1957; Rae et al., 2000, a severe immunodeficiency characterized not only by life threatening infections but also by autoimmune manifestations, such as inflammatory bowel disease (Singel and Segal, 2016). The most common cause of CGD, X-linked gp91*phox* deficiency (Segal, 1987; Teahan et al., 1987), is associated with a significantly reduced life expectancy. Of note, the most frequent causes of septicemia in patients with CGD are *Salmonella* species (van den Berg et al., 2009).

The genome-wide set of targeted mutations in mouse ES cells established by the KOMP, EUCOMM, and MirKO programs provides an opportunity to conduct systematic, large-scale gene function analysis in a mammalian system (Ayadi et al., 2012; White et al., 2013). Therefore, to uncover new pathways involved in host defense, we screened hundreds of knockout mouse lines for susceptibility to infection by inoculating mice generated through KOMP with an attenuated form of the intracellular pathogen, *S.* Typhimurium () In this study, we show that mice deficient in a previously uncharacterized gene, *bc017643*, are highly susceptible to *Salmonella* and *Listeria* infection and are impaired in their ability to control replication of either pathogen. We show that this is because *bc017643* encodes a gene, which we name *Eros* (*essential for reactive oxygen species*), that is necessary for the phagocyte respiratory burst. *Eros* is necessary for stable expression of the gp91*phox* and p22*phox* subunits of the cytochrome b558 heterodimer. Intriguingly, a plant ortholog of Eros, Ycf4, is necessary for expression of the subunits of photosystem I in chloroplasts, also an NADPH oxio-reductase. Collectively, our work identifies a novel and essential regulator of the phagocyte respiratory burst and points to an ancient and conserved function for a hitherto undescribed family of proteins.

## Results

### Mice deficient in the uncharacterized gene, *bc017643,* are highly susceptible to *Salmonella enterica* serovar Typhimurium but not *Citrobacter rodentium* infection

**Figure 1.**
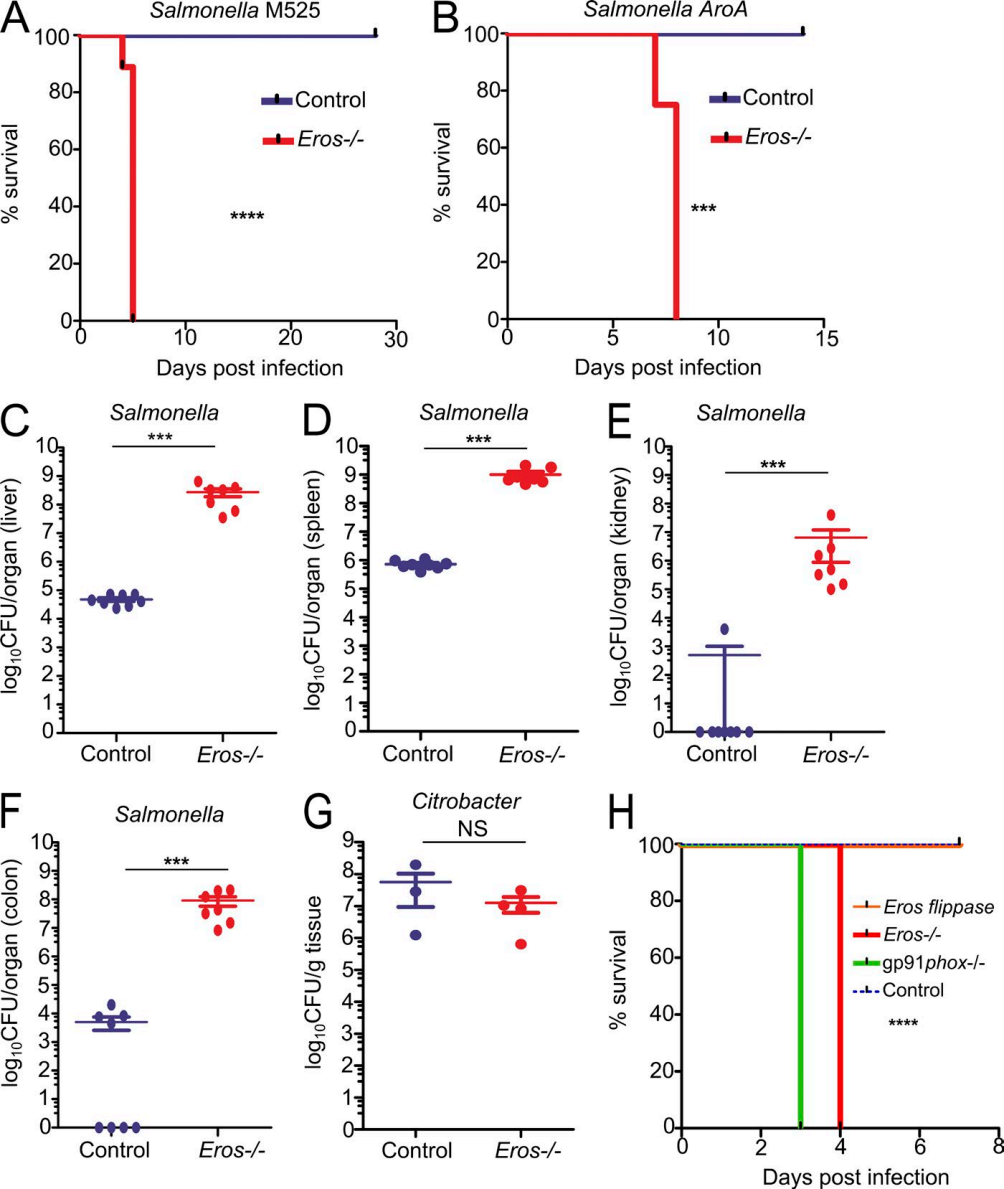
***Eros* is essential for host defense against *S.* Typhimurium.** (A and B) Survival of *Eros*^−/−^ mice after i.v. challenge with *S.* Typhimurium M525 (A) or after oral challenge with *S.* Typhimurium ΔaroA (B). Data in A show eight mice per group and are representative of greater than five independent experiments. Data in B show eight mice per group and are representative of two independent experiments. (C–F) Bacterial burden expressed as CFU per organ in liver (C), spleen (D), kidney (E), and colon (F) at day 4 after infection with *S.* Typhimurium M525. Eight mice were used in each group. Results are representative of greater than five independent experiments (G) *C. rodentium* burden in colon after oral challenge. Eight mice were used per group and data are representative of two independent experiments. (H) Survival of control (eight mice), *Eros*-deficient (five mice), gp91*phox*-deficient (four mice), and *Eros*-sufficient flippase mice (repaired *Eros* allele; eight mice) after infection with 5 × 10^5^ CFU of *S.* Typhimurium M525. Data are representative of two independent experiments. The P-value shown in H denotes the significant difference in survival between *Eros*-flippase and *Eros*-deficient mice. Error bars represent the SEM. ***, P < 0.001; ****, P < 0.0001. Data in A, B, and H were analyzed by Log-Rank test and data in C–G were analyzed by Mann-Whitney test.

Infections by facultative intracellular pathogens such as *Salmonella* cause major morbidity and mortality globally, but our understanding of host defense to such pathogens remains incomplete. To address this in a systematic fashion and to explore host susceptibility to infection more generally, we screened mice generated through the Wellcome Trust Sanger Institute Knockout Mouse Project for immunity to *Salmonella enterica* serovar Typhimurium (*S*. Typhimurium) challenge (White et al., 2013). Mice harboring a targeted mutation in *bc017643*, a previously uncharacterized protein-coding gene located on chromosome 11 (Fig. S1 A), were highly susceptible to *S*. Typhimurium, dying 4–5 d after intravenous inoculation with the attenuated *S*. Typhimurium M525 (Fig. 1 A). As we demonstrate that *bc017643* is crucial for the generation of a phagocyte respiratory burst in the innate immune system, we have renamed the gene *Eros*. *Eros*^−/−^ mice were also susceptible to the highly attenuated *S*. Typhimurium vaccine strain SL261 Δ*aroA* (Fig. 1 B), underlining the severity of the phenotype. The susceptibility of *Eros*^−/−^ mice to *Salmonella* infection was caused by a failure to control bacterial replication as they harbored 10^3^–10^4^ log-fold more CFU of *Salmonella* per gram of spleen or liver than control mice at day 4 after challenge. In addition, there was evidence of infection in the kidney and colon (Fig. 1, C–F), sites which are less heavily colonized by *S*. Typhimurium. *Eros^+/−^* heterozygous mice were not more susceptible to *S.* Typhimurium (Fig. S1 B). In contrast to the early lethality with *S*. Typhimurium infection, *Eros*^−/−^ mice were not more susceptible to the extracellular mucosal pathogen *Citrobacter rodentium* (Fig. 1 G and not depicted). Confirmation of the causative role of the previously uncharacterized *Eros* gene in driving susceptibility to *Salmonella* was provided by repairing the knockout allele and reintroducing *Eros* expression. This was accomplished by crossing the *Eros*-deficient mice with flippase-expressing mice to remove the FRT-flanked stop codon that generates the original null allele. This restoration of *Eros* abolished the susceptibility to *Salmonella* infection (Fig. 1 H).

### Eros is a transmembrane protein with homology to Ycf 4

**Figure 2.**
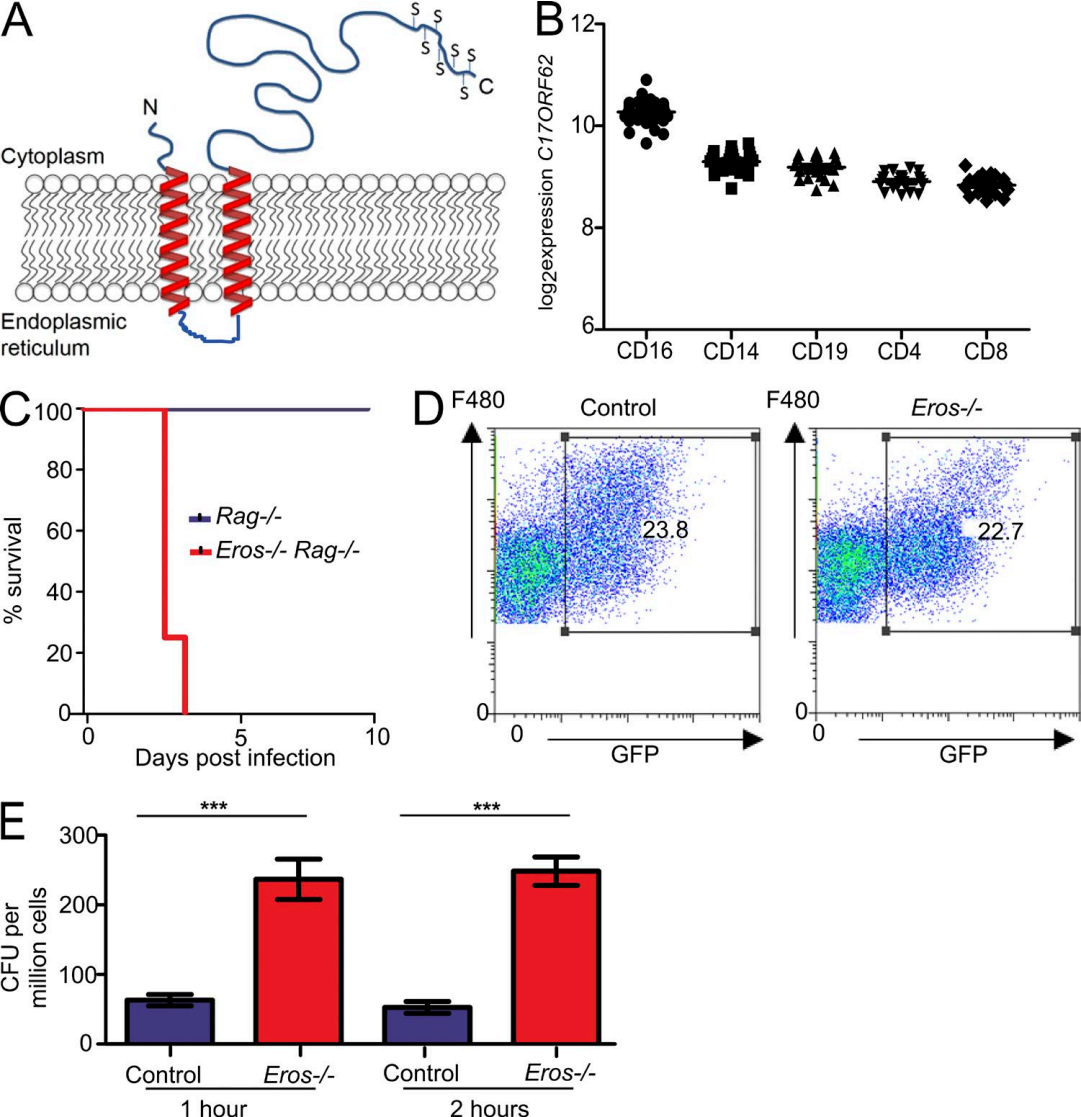
**Eros is a transmembrane protein, and *Eros*-deficient macrophages fail to kill *S.* Typhimurium.** (A) Schematic diagram of *Eros* structure based on prediction algorithms described in text. Serines at the C terminus are denoted by S. (B) Expression of *Eros* ortholog *C17ORF62* in peripheral blood cells of healthy volunteers by probe level expression on microarray. Each dot represents one individual. (C) Survival of *Eros*^−/−^ *Rag^−/−^* double-deficient mice and *Rag^−/−^* mice (eight mice per group) after i.v. infection with 10^6^ CFU of *S.* Typhimurium M525. Results are representative of three independent experiments. (D) Flow cytometric analysis of uptake of opsonized spi2-inducible GFP expressing *S.* Typhimurium by F480^+^ peritoneal macrophages. Results are representative of three independent experiments. (E) Killing of M525 *S.* Typhimurium by control or *Eros*^−/−^ peritoneal macrophages in a gentamicin protection assay. Macrophages from three independent mice were used in each group, and data are representative of three independent experiments. Error bars represent the SEM. ***, P < 0.001. Data in A were analyzed by log-rank test and data in E were analyzed by unpaired Student’s *t* test.

There is no published literature on murine *Eros*. The protein shares ∼89% sequence identity with human *C17ORF62* and is predicted to be a single domain protein and shares sequence homology with domain of unknown function (DUF) 4564. Sequence similarity searching using jackhmmer (Finn et al., 2011) identified orthologs of *Eros* in all vertebrates, as well as in some lower order animals such as poriferan sponges and sea urchins. This suggests that *Eros* has an ancient evolutionary origin. *Eros* also shares significant sequence similarity with the conserved plant protein Ycf4 (Boudreau et al., 1997; Krech et al., 2012), which participates in the assembly of the photosystem I complex (Boudreau et al., 1997). Phobius software (Käll et al., 2004) predicted an N-terminal transmembrane region comprising two transmembrane helices (residues 21–39 and 45–63) with the N- and C-terminal regions present within the cytoplasm and the two transmembrane helices separated by a short linker sequence of five residues (Fig. 2 A). The protein is also likely to contain a disordered C-terminal (residues 168–187) that is rich in serine residues (7 out of 20 residues). Databases of RNA and protein expression (European Bioinformatics Institute Expression Atlas and Immgen) show that *Eros* is highly expressed in cells of the innate immune system, particularly in neutrophils, monocytes, and macrophages. The protein has been detected in the phagosome of macrophages (Trost et al., 2009; Dill et al., 2015) and in dendritic cell endosomes (Nakamura et al., 2014). The human ortholog of *Eros*, *C17ORF62*, is also highly expressed in the immune system. Microarray analysis of separated blood subsets from healthy volunteers detected the highest expression in neutrophils and monocytes (Fig. 2 B). This is consistent with a proteomic analysis of separated human tissues that showed high expression of C17ORF62 in monocytes and CD4 T cells and B cells, although neutrophils were not evaluated in this study (Kim et al., 2014). Mass spectrometry data from human induced pluripotent stemcell–derived macrophages in our own laboratory showed that C17ORF62 protein expression was up-regulated after treatment with IFN-γ or a combination of IFN-γ and *S*. Typhimurium (Fig. S1, C and D). Consistent with high expression of the protein in monocytes, macrophages, and neutrophils, the severe phenotype associated with *Eros* deficiency was still evident when the mice were crossed to the *Rag*-knockout background (Fig. 2 C), confirming that abnormalities in the innate immune system alone could account for the susceptibility to *S*. Typhimurium.

### *Eros*-deficient macrophages fail to kill *S*. Typhimurium ex vivo

Flow cytometric analysis of cells from uninfected *Eros*^−/−^ mice demonstrated no abnormality in the number or frequency of any of the major immune cell subsets (Table 1). The normal function of macrophages (Vazquez-Torres et al., 2004), monocytes (Tam et al., 2008; Rydström and Wick, 2010), and neutrophils (Conlan, 1997) is required to control *Salmonella* replication. Accordingly, we tested the functional capabilities of peritoneal macrophages from *Eros*^−/−^ mice in vitro. *Eros*^−/−^ peritoneal macrophages were able to phagocytose bacteria apparently normally (Fig. 2 D) and electron microscopy showed that *Eros*^−/−^ macrophages could internalize bacteria into a *Salmonella*-containing vacuole (unpublished data). However, peritoneal macrophages from *Eros*^−/−^ mice showed a marked defect in the ability to control intracellular *Salmonella* replication 1–2 h after ex vivo infection, as measured by gentamicin protection assays (Fig. 2 E), complementing our observations in vivo. Although IL-12, IFN-γ, and TNF are all important for the control of *Salmonella,* there were no obvious defects in cytokine secretion by *Eros*^−/−^ BM-derived macrophages in response to live opsonized *S.* Typhimurium M525 (Table 2). Neutrophils have also been implicated as essential to control of *S.* Typhimurium (Conlan, 1997; Tam et al., 2008; Burton et al., 2014). We confirmed this in our model by using the anti-Ly6G antibody, 1A8, to deplete neutrophils. We then infected mice with *S.* Typhimurium M525 and measured bacterial burden in liver and spleen 72 h later. Control mice treated with 1A8 had higher counts (of the order of 1-log fold) than controls (Fig. S1, E and F), indicating that neutrophils do contribute to the control of *S.* Typhimurium. We also found that *Eros*-deficient neutrophils were unable to control *S.* Typhimurium replication. Using an RFP-expressing *S.* Typhimurium SL1344 strain, we showed that uptake of bacteria was equivalent as at 1 h after infection; the percentage of infected neutrophils was not different between control and *Eros*^−/−^ neutrophils. However, at 3 h, both the proportion of cells infected with *S.* Typhimurium (Fig. S1, G and H) and the mean fluorescence intensity (a surrogate marker of bacterial load) were higher in *Eros*-deficient cells.

**Table 1.** Normal immune cell numbers in *Eros^−/−^* mice

	Control (22 mice)	*Eros^−/−^* (18 mice)
**Cell number (spleen) × 10^−6^**		
Total splenocytes	50.67 ± 0.63	44.9 ± 0.45
F480^+^ macrophages	1.62 ± 0.11	1.639 ± 0.12
CD11c^hi^ dendritic cells	1.82 ± 0.01	1.83 ± 0.11
CD11c^hi^ CD8^+^ dendritic cells	0.46 ± 0.03	0.43 ± 0.03
Ly6C^hi^ monocytes	0.91 ± 0.07	0.75 ± 0.04
Neutrophils	2.56 ± 0.21	2.1 ± 0.09
**Cell number (blood) × 10^−6^/ml**		
Ly6C^hi^ monocytes blood	5.9 ± 0.7	4.3 ± 0.46
Ly6C^lo^ monocytes blood	1.9 ± 0.25	2.0 ± 0.20
Neutrophils	9.24 ± 0.46	8.3 ± 0.34

Mean cell number of the indicated subsets in the relevant organs ± SEM. Data were analyzed by Mann-Whitney test and represent pooled data from three independent experiments. There were no significant differences between control and *Eros^−/−^* mice in any subset studied.

**Table 2.** Cytokine/chemokine production by BM-derived macrophages from control and *Eros^−/−^* BM-derived macrophages 18 h after incubation with live *S.* Typhimurium M525

Cytokines	Control	*Eros^−/−^*
	*pg/ml*	*pg/ml*
TNF	8,596 ± 490	7,564 ± 463
IL-12p70	138 ± 15	100 ± 15
KC/GRO	6,736 ± 282	5,751 ± 482
IL-10	4.955 ± 182	5,245 ± 325
IL-6	5,497 ± 384	6,188 ± 571
IFN-γ	41 ± 2	43 ± 3

Values presented represent the mean cytokine production in culture supernatants ± SEM. Data were analyzed by unpaired Student's *t* test and are representative of three independent experiments. There were no significant differences between control and *Eros^−/−^* mice in any subset studied.

### The phagocyte respiratory burst is highly impaired in *Eros*-deficient neutrophils and macrophages

**Figure 3.**
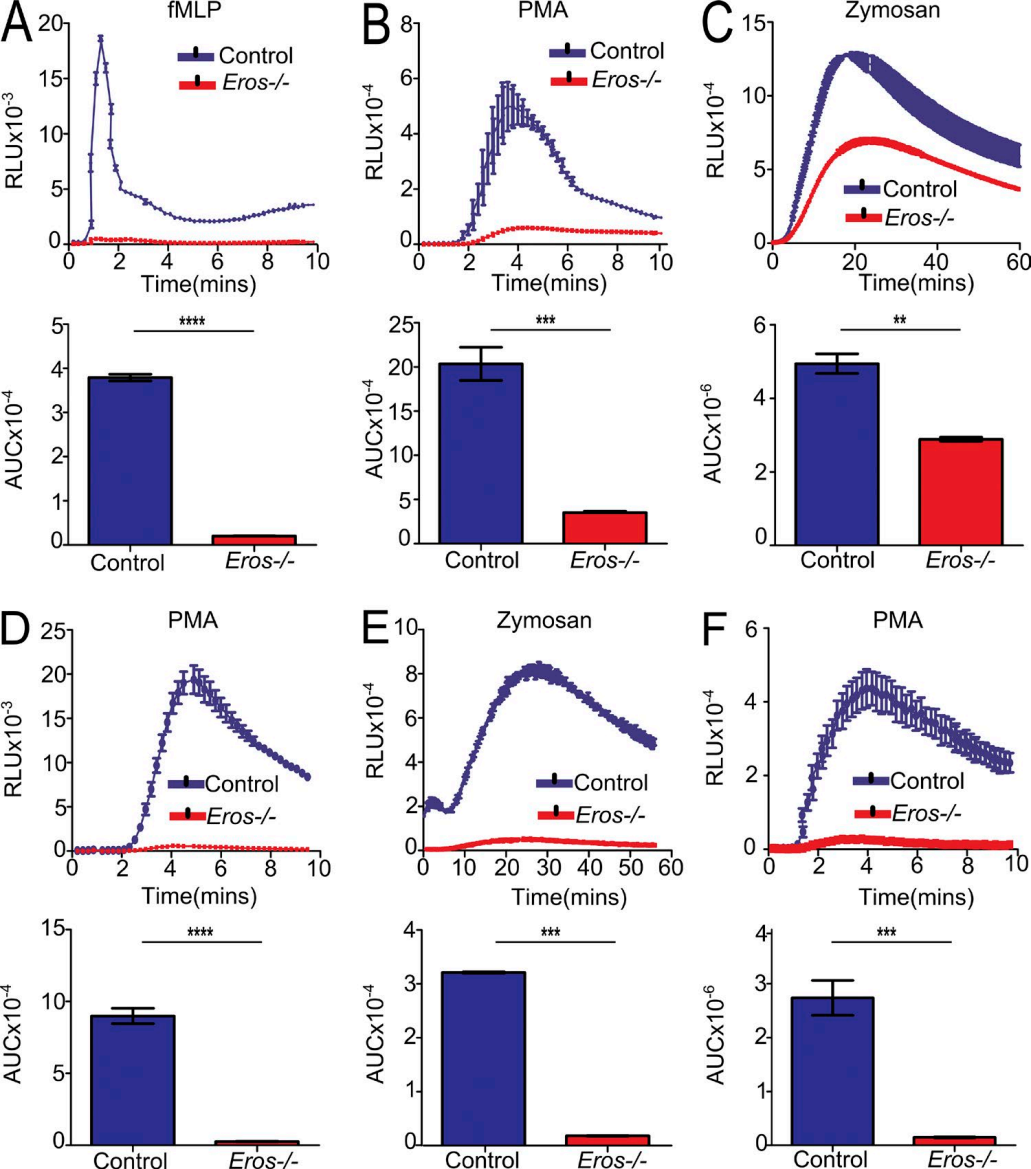
***Eros*^−/−^ neutrophils and macrophages have a severely impaired phagocyte respiratory burst in vitro.** (A–C) ROS production (three technical replicates, each control and *Eros*^−/−^ sample is pooled from two mice) measured in relative light units (RLU) by purified BM neutrophils from control or *Eros*^−/−^ mice in response to (A) fMLP, (B) PMA, or (C) zymosan. Data are representative of at least three independent experiments. (D and E) ROS production (replicates from BM derived from three individual mice, measured in RLU) by purified BM-derived macrophages from control or *Eros*^−/−^ mice in response to PMA (D) and zymosan (E). (F) ROS production by peritoneal cells from *Rag^−/−^* (three mice) or *Eros*^−/−^ *Rag^−/−^* mice (three mice) in response to PMA. Data are representative of at least three independent experiments. Area under the curve (AUC) was calculated for each sample. Error bars represent the SEM. **, P < 0.01; ***, P < 0.001; ****, P < 0.0001, analyzed by unpaired Student’s *t* test.

We noted that *Eros*-deficient mice had a very similar phenotype to that of mice deficient in components of the NADPH oxidase complex, including gp91*phox* (Nox2) and p22*phox* (Mastroeni et al., 2000; van den Berg et al., 2009; and Fig. S1, I and J). These mice, like *Eros*^−/−^ mice, are highly susceptible to *S.* Typhimurium but survive *C. rodentium* infection (Fattouh et al., 2013; and unpublished data). Testing this phenotype, we found that neutrophils from *Eros*^−/−^ mice had a highly impaired superoxide burst in vitro. This was most marked in response to the N-formylated peptide *N*-formylmethionyl-leucyl-phenylalanine (fMLP), to which the response was almost absent (Fig. 3 A). There was a 5–10-fold impairment of the response to the protein kinase C activator phorbol 12-myristate 13-acetate (PMA; Fig. 3 B), but a variable and slightly less marked defect in the response to zymosan (Fig. 3 C). The defect in the phagocyte respiratory burst was equally apparent using alternative luminescent indicators, such as lucigenin and DIOGENES (unpublished data). The phenomenon extended to other *Eros*^−/−^ phagocytes. BM-derived macrophages from *Eros*^−/−^ mice exhibited a severely impaired generation of superoxide in response to PMA (Fig. 3 D) and zymosan (Fig. 3 E), as did peritoneal macrophages (Fig. 3 F). To put the impaired phagocyte respiratory burst in context, we compared *Eros*^−/−^ neutrophils with mice lacking gp91*phox* or p22*phox*, the two essential membrane-bound components of flavocytochrome b558. These experiments confirmed that, in neutrophils, the phagocyte respiratory burst is highly impaired rather than totally absent in *Eros*^−/−^ mice (Fig. S2, A and B). The deficits observed in *Eros*^−/−^ BM-derived macrophages were even more severe than those in neutrophils, and their ROS generation was as impaired as that of gp91*phox*^−/−^ mice, including the ex vivo response to *S.* Typhimurium itself (Fig. S2, C–E). Consistent with some residual oxidase activity in neutrophils, comparison of *Salmonella* infection in *Eros*^−/−^ mice with those deficient in either gp91*phox* or p22*phox* showed that they had a slightly less severe defect in controlling bacterial replication than gp91*phox* or p22*phox*-deficient mice, which have no oxidase activity at all (Fig. S3 A).

### *Eros*^−/−^ mice exhibit other sequelae of a defective phagocyte respiratory burst

**Figure 4.**
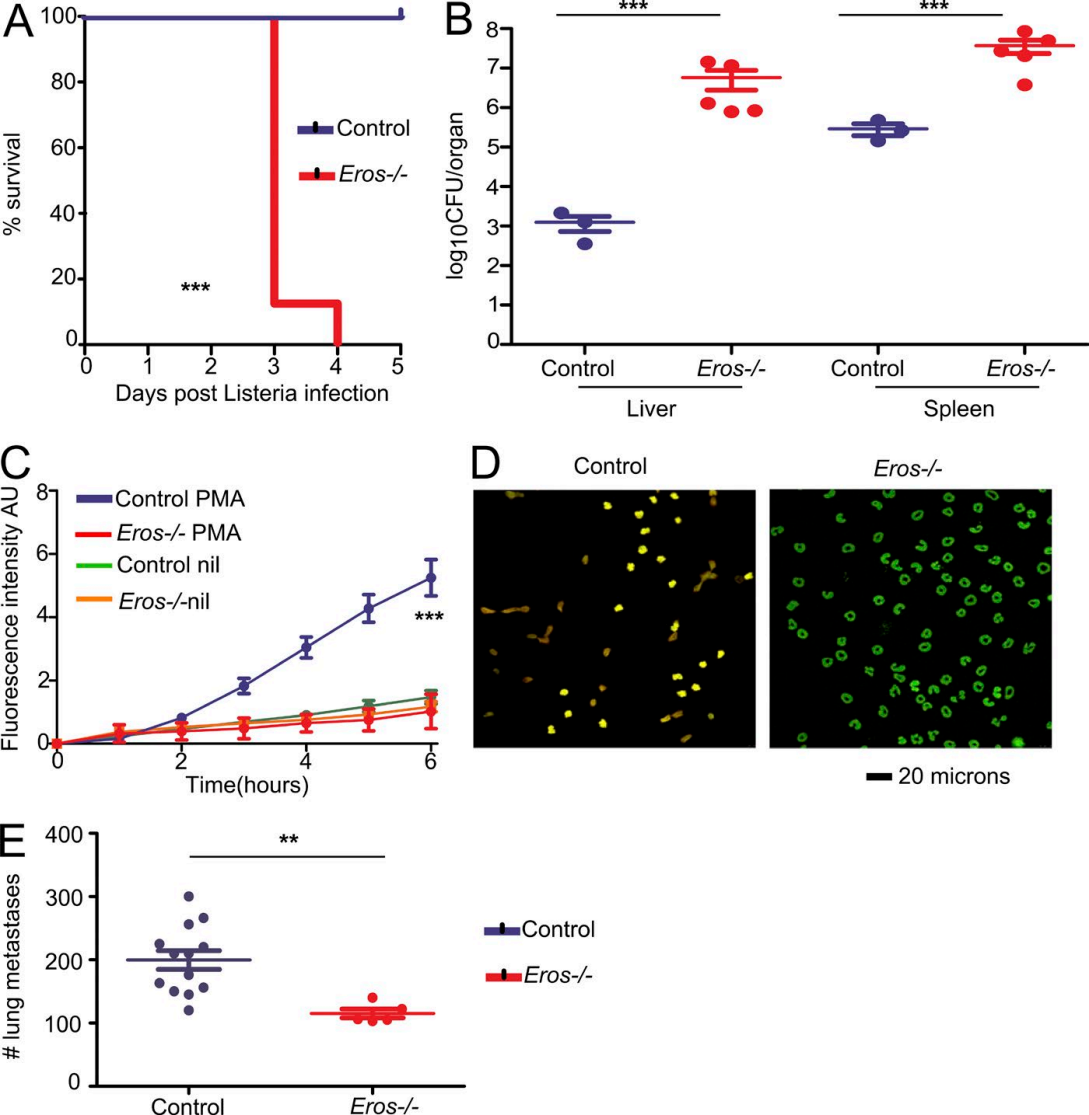
***Eros*^−/−^ mice are susceptible to *L. monocytogenes* infection and fail to form NETS but are resistant to melanoma metastasis.** (A) Survival of control (three mice) and *Eros*^−/−^ (five mice) mice after i.v. injection with *L. monocytogenes*. (B) Bacterial burden in liver and spleen of control and *Eros*^−/−^ mice 3 d after infection with *L. monocytogenes.* Data in A and B are representative of three independent experiments. (C) Neutrophil extracellular trap formation in response to PMA by control and *Eros*^−/−^ mice. NETosis is measured by absorbance of Sytox Green. (D) Representative staining of control and *Eros*^−/−^ neutrophils with sytox green and anticitrullinated histone 3 antibody. (E) Number of lung metastases in control or *Eros*^−/−^ mice 10 d after i.v. injection of B16-F10 melanoma cells. Error bars represent the SEM. **, P < 0.01; ***, P < 0.001. Data in A were analyzed by Log-rank test. Data in B and E were analyzed by Mann Whitney test. Data in C was analyzed by Tukey’s test. Data are representative of at least three independent experiments.

We reasoned that the consequences of Eros deficiency should extend to other processes that are dependent on an intact phagocyte respiratory burst, such as clearance of *Listeria monocytogenes* (Dinauer et al., 1997; Noubade et al., 2014). *Eros*^−/−^ mice were highly susceptible to *L. monocytogenes* infection (Fig. 4 A) and died within 5 d of infection, with high bacterial loads in liver and spleen (Fig. 4 B). Under certain specific circumstances, the generation of neutrophil extracellular traps (NETS) is also dependent on an intact phagocyte respiratory burst (Remijsen et al., 2011). NET production in response to PMA was markedly impaired in *Eros*^−/−^ mice (Fig. 4, C and D). We then asked whether *Eros* deficiency could ever be advantageous. The phagocyte respiratory burst can, for example, hinder tumor immunity. gp91*phox*, *Ncf1* (p47*phox*), and Rac2-deficient mice all exhibit fewer lung metastases than control mice in experimental melanoma models (Okada et al., 2006; Kelkka et al., 2013; Joshi et al., 2014). We challenged mice with B16 melanoma cells and demonstrated that *Eros*-deficient mice had significantly fewer lung metastases 10 d later (Fig. 4 E). This demonstrates that the influence of *Eros* extends to other biological processes in which the phagocyte respiratory burst is known to be important.

### *Eros*^−/−^ neutrophils and macrophages express very low levels of gp91*phox* and p22*phox* proteins

**Figure 5.**
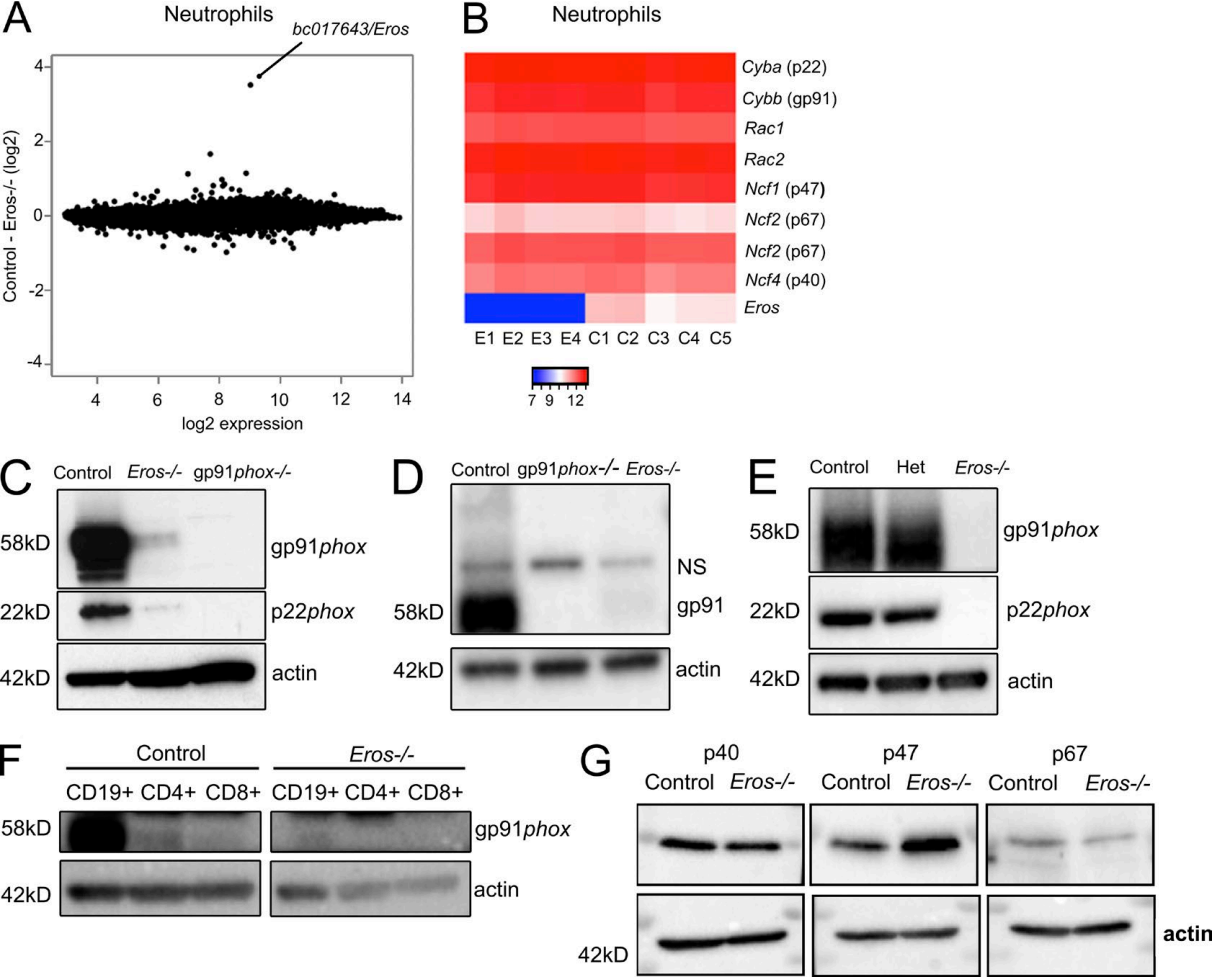
***Eros*^−/−^ mice have severely reduced expression of cytochrome b558 (gp91*phox*/p22*phox* heterodimer).** (A) mRNA microarray analysis of purified BM neutrophils from control (five individuals) and *Eros*^−/−^ (four individuals) mice showing *bc017643* (*Eros*) as the only statistically significant differentially expressed gene. 1 (B) Heat map of selected genes from A. Samples from individual Eros^–/–^ mice are denoted E1-E4 and those from control mice are denoted C1-C5. (C) gp91*phox* and p22*phox* expression in purified BM neutrophils from control, *Eros*^−/−^, and gp91*phox^−/−^* neutrophils. Data are representative of at least three independent experiments. (D) gp91*phox* expression in peritoneal macrophages from control, *Eros*^−/−^, and gp91*phox*^−/−^ mice. NS, nonspecific band. Data are representative of at least three independent experiments. (E) gp91*phox*and p22*phox* expression in BM-derived macrophages from control, *Eros*^−/−^, and Eros^+/−^ (het) mice. Data are representative of three independent experiments. (F) gp91*phox* expression in lymphocytes from control and *Eros*^−/−^ mice. Data are representative of two independent experiments. (G) Expression of the indicated cytoplasmic subunits of the phagocyte NADPH oxidase in neutrophils from control and *Eros*^−/−^ mice. Data are representative of three independent experiments.

The phenotypes we observed led us to examine whether *Eros*^−/−^ mice had abnormal expression of components of the phagocyte NADPH oxidase. *Eros* deficiency had no measurable effect on basal RNA expression in freshly isolated BM neutrophils or in whole spleen; the only differentially expressed gene identified by microarray analysis in *Eros*^−/−^ mice was *Eros* itself (Fig. 5 A and Fig. S4 A). In particular, there was no difference in the mRNA level of the genes encoding the subunits of the NADPH oxidase (Fig. 5 B). Expression of both the gp91*phox* and p22*phox* proteins was, however, markedly reduced but not entirely absent in *Eros*^−/−^ neutrophils. This was demonstrated by comparison of *Eros*^−/−^ neutrophils with those from gp91*phox*-deficient (*Cybb* knockout) mice where the protein was undetectable (Fig. 5 C). gp91*phox* and its membrane-bound partner, p22*phox*, are only stable as a heterodimer. Therefore, if one member of the heterodimer is weakly expressed, abundance of the partner protein is reduced (Segal, 1987; Pollock et al., 1995). Peritoneal lavage cells, IFN-γ–primed BM-derived macrophages and splenic B and T cells also exhibited very low levels of gp91*phox* and p22*phox* in *Eros*^−/−^ cells. (Fig. 5, D–F). In contrast, the cytoplasmic components of the NADPH oxidase complex, p47*phox*, p67*phox*, and p40*phox* were not significantly differentially expressed by Western blot (Fig. 5 G) or by mass spectrometry (unpublished data). gp91*phox* has several homologues which are highly expressed in other tissues. In particular Nox1 is highly expressed in the colon and endothelium, Nox3 is specifically expressed in the inner ear, and Nox4 is predominantly expressed in the kidney. Nox1-4 all use p22*phox* to form a stable heterodimer. Among members of the Nox family, the role of Eros protein seems to be specifically restricted to regulating the abundance of gp91*phox* (Nox2). We measured expression of Nox1 and Nox4 by Western blot. As expected, we saw the highest levels of Nox1 in the colon and high expression of Nox4 in the kidney. Both proteins were equivalently expressed between control and *Eros*^−/−^ mice (Figs. S3, B and C). Nox3 is highly expressed in the inner ear and Nox3-deficient mice exhibit a vestibular/balance defect characterized by head tilt, and therefore we could assess Nox3 using functional assays that were used to phenotype mice as part of the Knockout Mouse Project (White et al., 2013). p22*phox* is essential for the stabilization of Nox3 in the inner ear, and thus nmf333 mice, which carry a homozygous mutation in p22*phox*, exhibit similar balance defects (Nakano et al., 2008). Consistent with this data, p22*phox*-deficient mice show spontaneous trunk curl and head bobbing, and fail to exhibit a contact-righting reflex, whereas *Eros*^−/−^ mice do not show any of these vestibular defects (Fig. S3, D–F).

### *Eros* deficiency has very specific effects on gp91*phox* and p22*phox* expression

**Figure 6.**
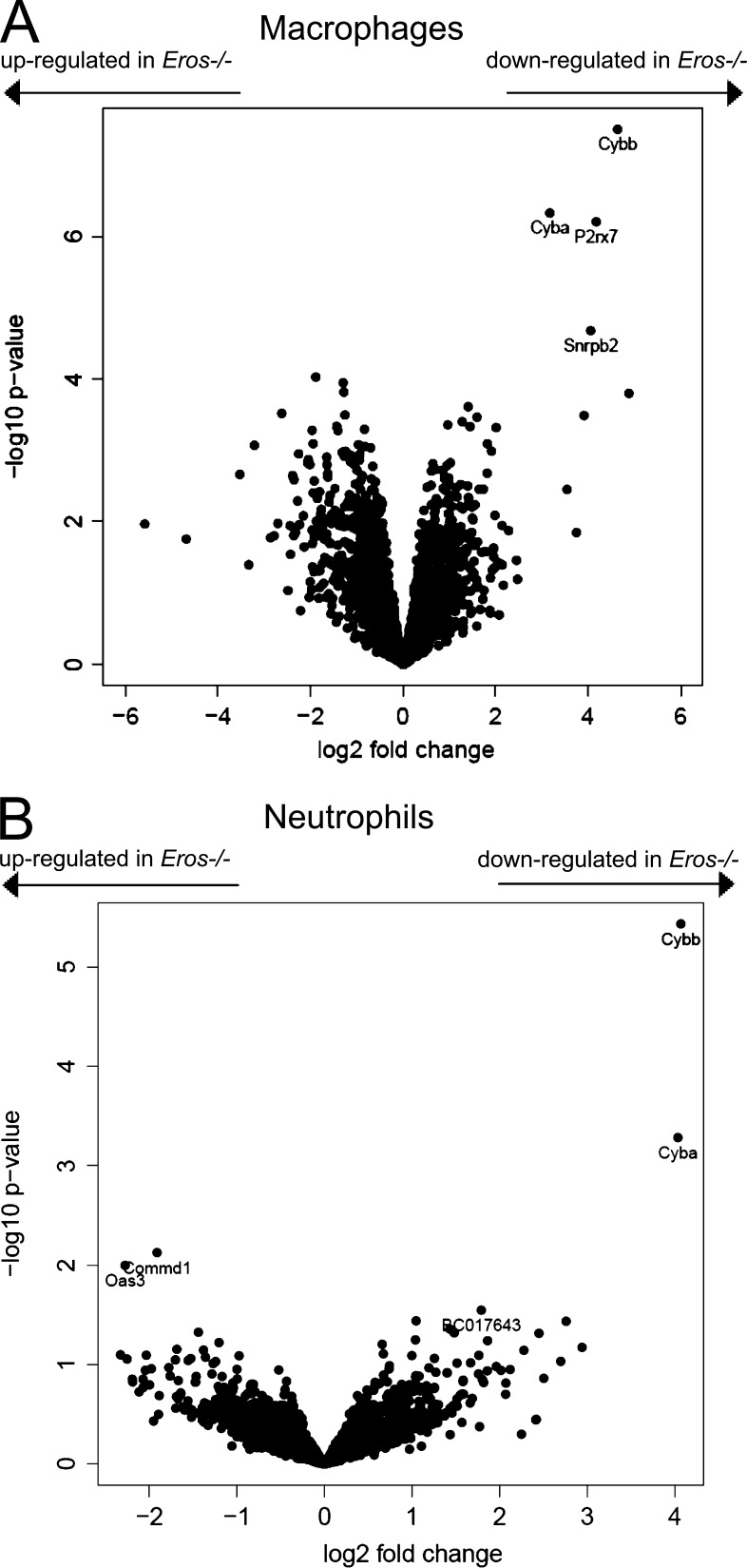
***Eros* deficiency has very specific effects on gp91*phox* and p22*phox* expression.** Volcano plots of label-free mass spectrometry from control and *Eros*^−/−^ innate immune cells. Data are shown for macrophages (A; four biological replicates for control and *Eros*^−/−^ mice) and neutrophils (B; three biological replicates for control and *Eros*^−/−^ mice). The volcano plots display statistical significance (Log_10_ P-value) versus the log_2_ fold change. *Cybb* and *Cyba* are the gene symbols for gp91*phox* and p22*phox* respectively.

Given that the components of the cytochrome b558 heterodimer were almost absent in *Eros*^−/−^ mice, we next asked whether the expression of other proteins was dysregulated in the innate immune system of *Eros*^−/−^ mice in the same profound manner as gp91*phox* and p22*phox*. We used label-free quantitative mass spectrometry to compare the whole macrophage proteome of control and *Eros*^−/−^ BM macrophages (Fig. 6 A). As expected, Eros protein was detectable in all samples from control cells but not those from *Eros*^−/−^ cells. In addition, gp91*phox* and p22*phox* were much less abundant in *Eros*^−/−^ cells, with many fewer unique peptides for both proteins detected in *Eros*^−/−^ neutrophils. Furthermore, *Eros* deficiency changed the abundance of a remarkably small subset of proteins, with the effects on gp91*phox* and p22*phox* the most profound. The log-fold change for gp91*phox* was 4.7. This was greater than for any other protein, except Eros, whereas the log-fold change for p22*phox* was 3.17. We performed a similar analysis in BM neutrophils (Fig. 6 B) and we found that only gp91*phox* and p22*phox* were differentially expressed between control and *Eros*^−/−^ cells. Other functional components of neutrophil defense were unaffected; for example, neither the amount of myeloperoxidase in neutrophil granules, nor elastase release, was affected in *Eros*^−/−^ cells (Fig. S4, B and C). Taking neutrophils and macrophages together, the only proteins that were significantly less abundant in both macrophages and neutrophils of *Eros*-deficient mice, were gp91*phox*, p22*phox*, and Eros protein itself.

### Eros localizes to the endoplasmic reticulum and co-immunoprecipitates with gp91*phox*

**Figure 7.**
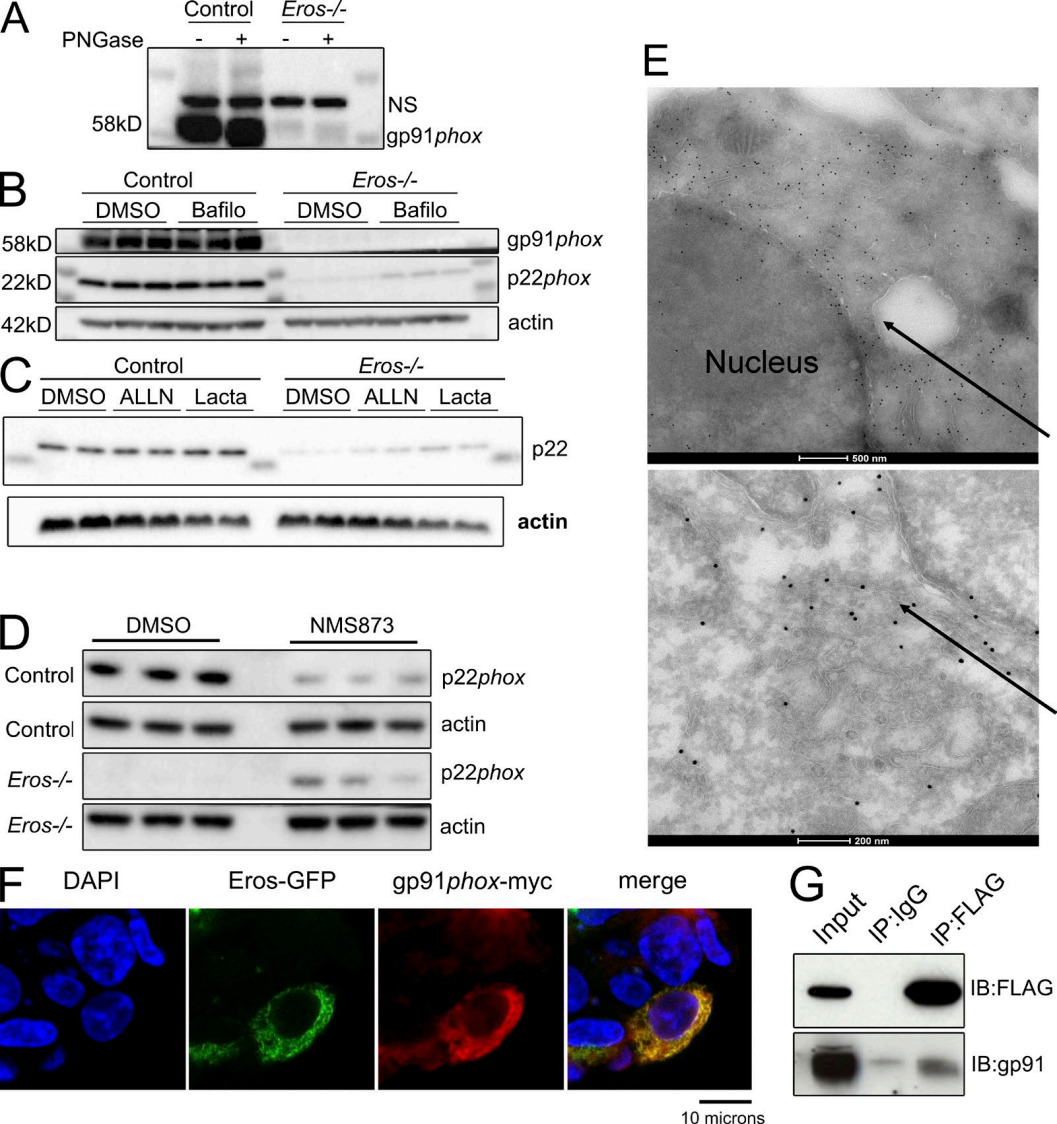
**Eros immunoprecipitates with gp91*phox* and also localizes to the endoplasmic reticulum.** (A) Western blot for gp91*phox* expression in BM-derived macrophages with or without treatment with PNGaseF. NS, nonspecific band. Data are representative of two independent experiments. (B and C) p22*phox* expression in control and Eros-deficient macrophages after incubation with bafilomycin (B) and ALLN and lactacystin (C) NMS873 (D). Data are representative of three independent experiments. (E) Electron micrographs of RAW264.7 cells transduced with *Eros*-FLAG, stained with anti-FLAG immunogold. Arrows indicate FLAG localization to the endoplasmic reticulum. Data are representative of three independent experiments. (F) Confocal microscopy of HEK 293T cells transfected with Eros-GFP and gp91*phox*-myc constructs. Data are representative of three independent experiments. (G) Immunoprecipitation of Eros-FLAG with a control antibody or anti-FLAG and blotting for FLAG or gp91*phox*, as indicated. Data are representative of three independent experiments.

We examined the mechanism underlying the low expression of the gp91*phox* and p22*phox* proteins in *Eros*^−/−^ cells. Defects in transcription, biosynthesis via glycosylation, and protein degradation of gp91*phox* and p22*phox* have all been shown to affect levels of the b558 flavocytochrome. An impact on translation is improbable given that mass spectrometry shows that *Eros* deficiency has an extremely specific effect on the macrophage and neutrophil proteome. An effect on biosynthesis is also unlikely, as there is no defect in glycosylation of the small amount of gp91*phox* protein present in *Eros*^−/−^ cells, which showed the same shift as control cells after treatment with PNGase (Fig. 7 A). Thus, Eros is likely to impact gp91*phox* and p22*phox* degradation, particularly as the abundance of gp91*phox* and p22*phox* protein is known to be controlled by endoplasmic reticulum–associated (ERAD) and proteasomal degradation (DeLeo et al., 2000; Chen et al., 2011, 2012). To test this, we incubated IFN-γ–primed BM macrophages with the proteasomal inhibitor MG132 and the p97 inhibitor NMS873, which blocks ERAD. We also treated such cells with the lysosomal inhibitor chloroquine and the autophagy inhibitor bafilomycin to determine if these pathways were driving accelerated degradation of gp91*phox* and p22*phox*. Chloroquine could not significantly rescue expression of either p22*phox* or gp91*phox* (unpublished data) in *Eros*^−/−^ cells. Bafilomycin could rescue p22*phox* expression to some extent, alone or in combination with MG132 (Fig. 7 B and Fig. S5 A). Both the proteasome inhibitor lactacystin and the calpain inhibitor ALLN were able to significantly rescue p22*phox* expression (Fig. 7 C and Fig. S5 B). Potent restoration of p22*phox* expression was observed with NMS873, consistent with data showing that this subunit is degraded by ERAD (Fig. 7 D and Fig. S5 C) and again consistent with increased degradation of p22*phox* in *Eros*^−/−^ mice. We were able to rescue gp91*phox* to a modest, though statistically significant, degree with ALLN and lactacystin, but not with the proteasome inhibitor MG132 or the ERAD inhibitor NMS873 (Fig. S5, D–F). In view of the fact that the rescue of gp91*phox* was minimal, the rescue of p22*phox* by ALLN, lactacystin, and NMS873 was probably the maximum that could be expected given that gp91*phox* is required to stabilize p22*phox* protein expression.

We next investigated the subcellular localization of *Eros* by transducing RAW264.7 cells with a lentiviral vector expressing recombinant *Eros* with an N-terminal FLAG-tag (Fig. S5, G and H) and found that Eros localized to the endoplasmic reticulum by electron microscopy (Fig. 7 E). gp91*phox* and p22*phox* are also known to localize to the endoplasmic reticulum of macrophages, as unassembled monomers. We found that by confocal microscopy, gp91*phox* and Eros co-localized in HEK293 cells (Fig. 7 F). As Eros, gp91*phox*, and p22*phox* all localize to the endoplasmic reticulum, we tested whether these proteins interact. Immunoprecipitation of Eros protein from RAW264.7 macrophages showed specific co-purification of gp91*phox* (Fig. 7 G), consistent with a role for this protein in stabilizing gp91*phox* expression. Thus, it is likely that *Eros,* in common with other proteins such as Nrros (Noubade et al., 2014), hsp70, and hsp90 (Chen et al., 2011, 2012), controls the degradation of gp91*phox* and p22*phox* in the ER through an interaction with, and stabilization of, the cytochrome b558 heterodimer.

## Discussion

Eros is a highly conserved, novel protein that is necessary for expression of the cytochrome b558 heterodimer and is therefore essential for the phagocyte respiratory burst and for host defense. Sequence analysis of Eros offered few clues to its function, though it does contain a domain of unknown function, DUF4564. Intriguingly, Ycf4, an ancient plant ortholog of Eros, also contains DUF4564 and also regulates the activity of an NADPH oxio-reductase, namely the ferredoxin oxio-reductase, photosystem I (PSI), which is essential for photosynthesis. The available data on Ycf4 offers some fascinating parallels with our observations on the behavior of Eros. Ycf4 was first identified in the unicellular green alga *Chlamydomonas reinhardtii* (Boudreau et al., 1997) and has also been studied in tobacco plants. (Krech et al., 2012). In Ycf4-deficient organisms, the components of PSI are not detectable at the protein level, despite normal mRNA expression (Boudreau et al., 1997; Krech et al., 2012). In addition, association of PSI transcripts with ribosomes was unaffected, implying that translation of PSI components was normal in Ycf4 mutant plants. Parallel with the ability of Eros to co-immunoprecipitate with gp91*phox*, Ycf4 interacts physically with PSI components. (Krech et al., 2012). We believe, then, that Eros is likely to play a similar role in the assembly of cytochrome b558 to that demonstrated for Ycf4 in algae and plants. Furthermore, our work implies an ancient conservation of function for the DUF4564 family of proteins in stabilizing the protein levels of oxio-reductase complexes. Eros is necessary for the stable expression of gp91*phox* and p22*phox*, most likely by acting as a chaperone. There is already strong evidence that the expression of gp91*phox* and p22*phox* is controlled at the level of protein degradation and that ER-resident chaperone proteins play a key role in this process. Studies on the mechanism of how gp91*phox* and p22*phox* assemble to form cytochrome b558 show that the unglycosylated gp91*phox* precursor and p22*phox* are synthesized in the ER and that gp91*phox* associates with p22*phox* before glycosylation in the Golgi apparatus. However, formation of the gp91*phox*-p22*phox* heterodimer is relatively inefficient and monomers that do not find a partner are rapidly degraded via the proteasome (DeLeo et al., 2000). There is precedence for the regulation of this interaction by chaperones. For instance, hsp70 and hsp90 play reciprocal roles in cytochrome b558 stability (Chen et al., 2011, 2012). Hsp90 stabilizes gp91*phox* expression whereas hsp70 facilitates its degradation via the hsp70-regulated ubiquitin ligase, CHIP (Chen et al., 2012). Nrros (negative regulator of reactive oxygen species) is also involved in cytochrome b558 stability, associating with gp91*phox* to facilitate its degradation (Noubade et al., 2014). Deficiency of Nrros in mice causes increased expression of the gp91*phox*-p22*phox* heterodimer with a concomitant increase in the magnitude of the phagocyte respiratory burst that has beneficial effects for clearance of bacterial infection but detrimental in that it leads to a more severe disease course of experimental allergic encephalomyelitis (Noubade et al., 2014). Eros is necessary for the stability of gp91*phox*-p22*phox* heterodimer and its deficiency has very specific effects on the abundance of these proteins, without affecting expression of their mRNA. Furthermore, gp91*phox* and Eros co-localize in the ER, and we have shown that these two proteins interact, as they can co-immunoprecipitate with each other. In addition, partial rescue of the expression of gp91*phox* and particularly p22*phox* can be achieved with inhibitors of the proteasome. Collectively, these data indicate that Eros plays a central role as a chaperone critical for expression of gp91*phox* and p22*phox* and hence for the cytochrome b558 heterodimer.

A fuller understanding of the phagocyte respiratory burst is not only necessary for the study of immunity to infection. CGD patients are more prone to a variety of autoimmune manifestations including inflammatory bowel disease and systemic lupus erythematosus (SLE; Singel and Segal, 2016), whereas polymorphic variation in components of the phagocyte NADPH oxidase machinery can play a role in the pathogenesis of polygenic autoinflammatory disease. Coding variants in the cytosolic p67*phox* subunit (*NCF2*) are associated with increased SLE (Jacob et al., 2012), and this is consistent with data suggesting that gp91*phox* deficiency predisposes to lupus in both mouse and man (Cale et al., 2007; Campbell et al., 2012). Aside from SLE and inflammatory bowel disease, genetic variation in p40*phox* (*NCF4*) has also been implicated in atopic dermatitis and ankylosing spondylitis. The lack of gp91*phox* and p22*phox* seen in *Eros*^−/−^ mice is certainly sufficient to cause abnormalities that extend beyond immunity to infection. We found that Eros deficiency influenced the formation of neutrophil extracellular traps and the host response to metastasis. The latter result is particularly intriguing as mice deficient in other components of the respiratory burst apparatus are also resistant to metastasis in mouse models (Okada et al., 2006; Kelkka et al., 2013; Joshi et al., 2014).

Hence, *Eros* is a novel and essential component of the phagocyte respiratory burst and is necessary for effective in vivo responses to common pathogens and the host response to tumor metastasis.

## Materials and methods

### Mice

*bc017643/Eros* knockout mice were generated using a tm1a (KOMP) knockout-first approach. Gene targeting was performed as part of the International Knockout Mouse Consortium (Skarnes et al., 2011). Targeted ES cells were selected for neomycin resistance and β-galactosidase expression, and the *bc017643^tm1a(KOMP)^* allele structure was confirmed by long-range PCR and sequencing. A single-integration event was confirmed by neomycin-copy number analysis with quantitative RT-PCR (qRT-PCR). The *bc017643^tm1a/tm1a^* mouse line was derived from the EPD0079_5_A11 ES cell clone and maintained on a C57BL/6N genetic background. The care and use of all mice was in accordance with UK Home Office regulations (UK Animals Scientific Procedures Act 1986). The mice were maintained in specific pathogen–free conditions and matched by age and sex within experiments.

### *S.* Typhimurium challenge

Eight *Eros*^−/−^ mice and eight C57BL6/N control mice aged 6–8 wk were infected i.v. with 0.2 ml *S.* Typhimurium M525 (phoN::tetC) containing 5 × 10^5^ CFU of bacteria in sterile PBS (Sigma-Aldrich). They were monitored in accordance with the protocol used for pathogen screening in the mouse genetics project (White et al., 2013). At various time points as indicated in the main text, mice were sacrificed and the spleen, liver, kidneys, blood, and cecal contents were harvested. Bacteria were enumerated by serial dilution and plating onto agar plates (Oxoid). Mice were weighed daily throughout the infection and were culled if they lost >20% of their starting weight in accordance with UK Home Office regulations (UK Animals Scientific Procedures Act 1986). In some specific experiments, mice deficient in gp91*phox* (*cybb*) or p22*phox* (*cyba*) were also included in the experiment. The procedure for infection with the *S.* Typhimurium SL1344 Δ*aroA* mutant was as described above except that on the day of infection, the mice were infected orally with 10^9^ CFU of bacteria. The procedure for infection with *L. monocytogenes* was as described above except that mice were infected with 0.2 ml containing 5 × 10^5^ CFU of *L. monocytogenes.* Statistical differences in survival between groups of mice were analyzed by log-rank test and differences in bacterial burden by Mann-Whitney test using the GraphPad Prism statistical package.

### *C. rodentium* challenge

Eight female *Eros*^−/−^ mice and eight female C57BL6/N control mice were infected orally with 0.2ml of *C. rodentium* ICC180 (10^9^ CFU) at 6 wk of age and monitored over 28 d. Every 2–3 d, faeces were collected from each mouse and the bacterial burden was enumerated by serial dilutions and plating onto agar plates. At day 14 after infection, four *Eros*^−/−^ and four control mice were sacrificed. The spleen, liver, cecal patch, cecum, cecal contents, and the last 6 cm of colon were extracted and the bacterial burden in the tissue or gut contents was assessed by serial dilution and plating onto agar plates. At day 28 after infection, the remaining mice were sacrificed and the day 14 after infection procedures were repeated. Mice are weighed daily throughout the infection. Statistical differences in bacterial burden were analyzed by Mann-Whitney test GraphPad Prism statistical package.

### Microarray hybridization and data analysis

Human peripheral blood samples were obtained from healthy volunteers (with ethical approval from NRES Committee East of England-Cambridge Central [refs: 08/H0308/176, 04/023, and 08/0306/21]). Neutrophils were obtained by lysing the pellet obtained after centrifugation over Histopaque 1077 (Sigma-Aldrich), then by positive selection using CD16^+^ MicroBeads (Miltenyi Biotec). Monocytes and CD4^+^ T cells were obtained by sequential positive selection using CD14^+^ and CD4^+^ MicroBeads (Miltenyi Biotec) and B-cells and CD8^+^ T cells were obtained by sequential positive selection using CD19^+^ and CD8^+^ MicroBeads (Miltenyi Biotec). mRNA for both mouse and human samples was extracted from cell lysates using RNEasy or AllPrep Mini kits (QIAGEN). RNA integrity was assessed by capillary electrophoresis using a BioAnalyser 2100 (Agilent Technologies) and its concentration was determined using a NanoDrop ND-1000 spectrophotometer. 200 ng total RNA was then labeled using the CONTROL Sense Target labeling kit (Affymetrix) and hybridized to either the Mouse Gene 1.0 ST arrays (mouse samples) or the Human Gene 1.1 ST array (human samples) according to the manufacturer's instructions. After washing, arrays were scanned using either a GS 3000 scanner or a GeneTitan instrument (Affymetrix). The resulting raw microarray data were normalized and summarized using the rma function of the oligo package in BioConductor. Human samples were hybridized across several batches; batch effects were removed using the ComBat algorithm with leukocyte subset as a covariate. Differentially expressed genes were identified using the limma package. Differential expression was defined as fold changes >1.5-fold that were statistically significant after correction for multiple testing by setting the false discovery rate to 5%.

### Bacterial uptake assay

Peritoneal cells were elicited from uninfected control or *Eros*^−/−^ mice with 5 ml of PBS at 4°C. Cells were centrifuged at 1,200 rpm for 5 min in an Allegra X-15R centrifuge (Beckman Coulter). The pellet was red cell lysed by resuspension in lysis buffer containing 0.15 M ammonium chloride and 0.01 M sodium hydrogen carbonate. The cells were washed with PBS and resuspended in Optimem (Gibco) at 2 × 10^6^ cells per well in a 24-well plate. *S.* Typhimurium SL1344 PssaG::GFP (which express GFP when Spi2 is active) were resuspended in PBS and incubated with 10% fresh mouse serum from control mice for 20 min at room temperature. They were added at 50 CFU per cell to the peritoneal cells and after 40 min, cells were harvested by gentle scraping, placed on ice, and incubated with anti–F480-PE (eBioscience) for 30 min at 4°C. The cells were washed in FACS buffer (PBS, 5% FBS, and 0.05% sodium azide) at 4°C and resuspended in PBS. FACS analysis was performed on a Cyan (Dako) or LSR-II (BD) flow cytometer. Peritoneal cells comprised 40–50% F480^hi^ macrophages and ∼50–60% B-1 B cells in all strains studied. In some experiments, involving neutrophils a *S.* Typhimurium SL1344 PssaG::RFP strain was used.

### In vitro bacterial killing assay

Macrophages were isolated by peritoneal lavage of control or *Eros*^−/−^ mice into PBS at 4°C. They were resuspended in Optimem (Gibco) at 2 × 10^6^ cells per well in a 24-well plate and incubated at 37°C overnight. Nonadherent cells were removed and the cells were incubated with *S.* Typhimurium M525 (phoN::tetC) opsonized with fresh autologous mouse serum at a ratio of 50 CFU per cell. After 1 h, extracellular bacteria were lysed by the replacement of the media with Optimem containing 50 µg/ml of gentamicin for 30 min. The solution was then replaced by one containing Optimem supplemented with 5 µg/ml gentamicin and cells were lysed 1 or 2 h later in double de-ionized water. Lysates were plated overnight on LB/ampicillin plates (Oxoid) to assess bacterial killing.

### Western blot for p47*phox*, p67*phox*, and p40*phox*

Control and *Eros*^−/−^ murine BM neutrophils (5 × 10^6^) were pelleted and resuspended in 100 µl of ice-cold lysis buffer (20 mM Tris-HCl, pH 7.5, 150 mM NaCl, 1 mM EDTA, 1 mM EGTA, 1% Triton X-100, 2.5 mM sodium pyrophosphate, 1 mM β-glycerophosphate, 1 mM PMSF, 1 mM sodium fluoride, 2 mM sodium orthovanadate, 10 µg/ml leupeptin, and protease inhibitor tablet Complete Mini Cat 11836170001 [Roche]) for 20 min on ice. Samples were briefly sonicated and the Triton insoluble fraction was spun down for 10 min at 10,000 *g* at 4°C. The supernatant was transferred to a fresh tube and the protein concentration was determined by the BCA (Bio-Rad Laboratories) protein assay kit, according to the manufacturer’s instructions. Samples were mixed with 5xSDS sample buffer and incubated at 95°C for 5 min 15 µg of protein from each sample was loaded on a 12% SDS-polyacrylamide mini-gel, and then subjected to electrophoresis at 150V. Precision Plus Protein Dual Color standards (10–250 kD; Bio-Rad Laboratories) were used as molecular weight markers and the gel was stopped when the 50-kD band reached within 0.5 cm of the bottom of the gel. The gel was washed with transfer buffer (25 mM Tris, 200 mM glycine, 0.2% SDS, and 20% methanol) blotted onto nitrocellulose membrane (N+ bond; GE Healthcare) using a semi-dry transfer at 150 A for 90 min. After transfer of the electrophoresed proteins, the membrane was blocked with 5% milk nonfat dry milk powder dissolved in Tris-HCL buffered saline (pH 8.0) containing 0.1% (wt/vol) Tween-20 for 1 h at room temperature. The membrane was immunoblotted with primary antibody, anti-p40*phox* pAb Upstate 07–501 (1:2,000), anti-p47*phox* pAb Upstate 07–500 (1:4,000), and anti p67*phox* pAb Upstate 07–502 (1:500) in Tris-HCL buffered saline (pH 8.0) containing 0.1% (wt/vol) Tween 20 at 4°C overnight.

### Western blot for gp91*phox*, p22*phox*, and Rac

Whole-cell extracts were prepared using a low-salt lysis buffer extracts (150 mM NaCl and 0.1% NP-40) as previously described (Pardo et al., 2010). Samples were separated by PAGE (NuPAGE Bis-Tris gels; Life Technologies) and analyzed by Western blotting. The following antibodies were used: gp91*phox* (sc-130543; Santa Cruz Biotechnology, Inc.), p22*phox* (sc-20781; Santa Cruz Biotechnology, Inc.), β-actin (Abcam, ab75186), and Rac1/2/3 (PA5-17159; Thermo Fisher Scientific). Blots were developed using ECL Prime reagents (GE Healthcare) and Lumigen TMA-6. Lysis in RIPA buffer produced similar results. 

### Experimental metastasis assay

Murine metastatic melanoma B16-F10 cells (ATCC) were cultured in DMEM (Lonza) supplemented with 10% FBS, 2 mM glutamine, and 100 IU/ml penicillin/streptomycin. Cells in log-phase growth were harvested and resuspended in PBS, and 5 × 10^5^ cells (in 0.1 ml) were injected into the tail vein of 6–8-wk-old female control and *Eros*^−/−^ mice. The animals were sacrificed 10 d later, and the lungs removed and rinsed in PBS. The number of metastatic foci were counted in a blinded fashion under a dissecting microscope. The data are reported as total number of metastatic foci per mouse. Any single value outliers from each genotype were identified using the Grubbs’ test (α = 0.05) and statistics were performed using the Mann-Whitney *t* test.

### Co-immunoprecipitation experiments

RAW macrophages expressing *Eros*-FLAG were lysed in 0.1% NP-40 lysis buffer containing 150 mM NaCl using a Dounce homogenizer. Cell extracts were incubated with anti-FLAG M2 antibody (F1804; Sigma-Aldrich) covalently coupled to Dynal Protein G magnetic beads (Invitrogen) for 90 min at 4°C. Beads were washed with 10 vol IPP150 buffer, and bound proteins were eluted by incubation in 1x LDS loading buffer (Life Technologies) at 70°C for 10 min, followed by reduction with 50 mM DTT. Proteins were separated by SDS-PAGE and transferred to nitrocellulose membranes. Membranes were blocked with 5% nonfat milk in PBS-0.1% Tween-20. All antibody incubations were done in this buffer with primary antibody recognizing gp91*phox* (sc-130543; Santa Cruz Biotechnology, Inc.). Secondary HRP-coupled anti–mouse, anti–rabbit, anti–goat antibodies (GE Healthcare), or HRP-Protein G (Millipore) were used. Detection was performed with ECL Plus (GE Healthcare).

### Purification of neutrophils

6–10-wk-old mice were sacrificed and BM was obtained by flushing femurs and tibias with PBS supplemented with 0.5% BSA at 4°C. The cells were resuspended in PBS at 4°C plus 0.5% BSA. Neutrophils were isolated using a Neutrophil Isolation kit (Miltenyi Biotec) used according to the manufacturer’s instructions.

### Extracellular DNA extrusion (NETosis assays)

BM-derived neutrophils were isolated as described above and were resuspended into IMDM media with 5 µM Sytox Green extracellular DNA dye (Life Technologies), with or without 20 nM PMA (Sigma-Aldrich), and seeded onto optical microplates (BD). To quantify the kinetics of NET formation, total fluorescence was measured hourly using a VICTOR3 Multilabel Reader using Wallac 1420 Workstation v3.00 software (PerkinElmer) and subtracted by t = 0 measurements. The presence or absence of NETs was confirmed visually by fluorescence microscopy. Experiments represent the mean ± SEM from three independent experiments conducted in duplicate. Statistical analysis was conducted by one-way analysis of variance and Tukey's test for significance.

### Confocal microscopy for NETosis assays

Samples were fixed by bringing the cell solution to 4% (vol/vol) paraformaldehyde (Thermo Fisher Scientific) in Dulbecco's PBS (Sigma-Aldrich). Samples were blocked in 2% BSA and 5% goat serum (Sigma-Aldrich) in PBS. Rabbit Histone H3 (citrulline R2+R8+R17) antibody (ab5103; Abcam) was used to immunostain for citrillinated histones. The primary antibody was incubated overnight at 4°C used at a dilution of 1/500 in PBS supplemented with 2% BSA and 5% goat serum. Anti–rabbit Alexa Fluor 568 (A11011; Invitrogen) was used as a secondary antibody. It was used at a dilution of 1/500 and incubated for 1 h at room temperature. Fluorescence microscopy images were taken using a Leica TCS SPE confocal microscope on a 63×/1.40–0.60 oil-immersion objective using Leica Application Suite Advanced Fluorescence v2.7 software (Leica Microsystems). Cells that had extruded DNA were marked yellow as a result of dual staining with both the Sytox green and the anticitrullinated histone antibody.

### Assessment of the phagocyte respiratory burst

To assess the kinetics of the phagocyte respiratory burst, purified neutrophils or macrophages at a dilution of 10^7^ per ml were warmed to 37°C for 15 min. 150 µl of neutrophils were preincubated for 3 min with luminol (2 µM) and horseradish peroxidase (62.5 IU/ml) before being stimulated with PMA (200 ng/ml; Sigma-Aldrich), fMLP (16 mM; Sigma-Aldrich), zymosan (100 µg/ml; Sigma-Aldrich), arachidonic acid (10 µM), or opsonized *Salmonella* Typhimurium M525 (50 bacteria per cell, opsonized for 20 min at 37°C with 10% fresh mouse serum). Light emission was assessed using a Berthold MicroLumat Plus luminometer (Berthold Technologies). fMLP and PMA were added through the injection port. Data output is in relative light units per second. In some experiments, alternative chemiluminescent substrates were used. The procedure was the same except that DIOGENES (National Diagnostics CL-202 used according to manufacturer’s instructions) or lucigenin (100 µM) were used in place of luminol. Curves shown are constructed using three technical or biological replicates where indicated. Area under the curve (AUC) was calculated using GraphPad Prism, and values were compared using an unpaired Student’s *t* test for each condition.

### Cloning of N-terminally FLAG-tagged *Eros/bc017643*

*Eros/bc017643* was N-terminally FLAG-tagged by cloning into a modified pHRSin lentiviral vector (a gift from Paul Lehner, CIMR, Cambridge University, Cambridge, England, UK; the vector was originally made in the laboratory of Y. Ikeda). In brief, bc017643 coding sequence was amplified from a plasmid template (MG201751; Origene) using the primers listed below. The amplicon was cloned into the pHRsin FLAG-tag vector using BamHI and NotI restriction sites. For the empty vector control, a modified version of pHRsinUbEm was made by ligating the annealed oligonucleotide pair into the vector using BamHI and NotI. Primers used for cloning were as follows: 5′-GGATCCTATATGCAGGTGGAGAC-3′ (bc017643_F_BamHI) and 5′-GCGGCCGCTCAACTCTGGCCACCAG-3′ (BC017643_R_NotI). Oligonucleotides used for making linker sequence were as follows: 5′-GATCCGTCGACGAATTCTGACTAA-3′ and 3′-GGCCTTAGTCAGAATTCGTCGAC-5′.

### Production of lentivirus and transduction of RAW264.7 cells

1 d before transfection, HEK293T cells were plated in T75 flasks to reach 40–50% confluency the next day. Cells were then transfected using polyethylenimine (PEI) at ratio of 3:1 (PEI:DNA). PEI was mixed with 1.6ml Optimem (Life Technologies), before addition of the packaging vectors pSPAX2, pMD2G, and pHRSin_*bc017643/Eros*_FLAG or pHRSin_Empty_Vector at a ratio of (3:1:4), using 7 µg of lenti-vector. The solution was gently mixed before incubating at room temperature for 15 min. The solution was then topped up to 12.5 ml with warm, complete media. All media was then removed from the flask of HEK293T cells before the addition of the transfection-ready media. Transfected HEK293T cells were checked for GFP positivity the following day but left undisturbed for 24 h. At 48 h, virus containing media was then collected and syringe-filtered through a 0.8-µm filter onto RAW 264.7 cells at a confluency of 30–40%. 12.5 ml of media was then replaced onto the transfected HEK cells and the process was repeated again the next day. Transduction efficiency of RAW 264.7 cells was determined by assaying for GFP positivity by flow cytometry on a FACSAria II (BD). Transduction efficiency was consistently between 90 and 95%.

### Flow cytometric analysis

Spleens were homogenized by manual disruption and red cells were lysed as described above. The cells were resuspended at 4°C in FACS buffer (PBS, 5% FBS and 0.1% sodium azide). Antibodies were incubated with cells for 30 min at 4°C at the indicated dilution and washed with FACS buffer before resuspension in fresh FACS buffer. Samples were analyzed on a Cyan flow cytometer (Dako) or an LSR-II flow cytometer (BD). The antibodies used were as follows: anti-Ly6G (48–5931-82; eBioscience; dilution 1/200), anti-CD11c (17–0114-81; eBioscience; dilution 1/200), anti-CD19 (48–0193-82; eBioscience; 1/200), anti CD11b (17–0112-82 or 45–0112-80; eBioscience), anti-CD8a (11–0081-82; eBioscience; 1/400 dilution), anti-CD4 (558107; BD), anti-F4/80 (12–4801-82; eBioscience; 1/200), anti-CD115 (12–1152-82; eBioscience; 1/250), anti CD45.1 (25–0453-82; eBioscience; 1/200), anti-CD45.2 (17–0454-81; eBioscience; 1/200), and anti-Ly6C (53–5932; eBioscience; 1/200).

### BM-derived macrophages

BM was harvested from the femurs and tibias of 6–10-wk-old control and *Eros^−/−^* mice into RPMI (Sigma-Aldrich), supplemented with 10% FBS, penicillin, and streptomycin at the concentration described above. Red cells were lysed with ammonium chloride lysis buffer as described above and grown in 100 × 20 mm Petri dishes at 4 × 10^6^ per plate, and grown in 10 ml RPMI medium as described above supplemented with 10% L929 cell supernatant (a gift from S. Mukhopadhay, Wellcome Trust Sanger Institute, Hinxton, England, UK). At day 3, the cultures were supplemented with an additional 10 ml of the same medium. In some cases, 10 ng/ml of recombinant mouse IFN-γ (PeproTech) was added overnight on day 5. On day 6, adherent cells were harvested by gentle scraping and were typically 80–90% F480^hi^ by flow cytometry.

### Analysis of cytokines in culture supernatants or serum

BM-derived macrophages were prepared as described above and 2 × 10^5^ cells in 100 µl of complete RPMI medium were plated in flat bottomed 96-well tissue culture plates (Cellstar). They were cultured alone or in the presence of live *S.* Typhimurium M525 (phoN::tetC) at 50 CFU per cell. Supernatants from stimulation of BM-derived macrophages were harvested at 18 h. Alternatively, serum was harvested after euthanasia of mice and subsequent cardiac puncture. Supernatants and serum were analyzed for the presence of the cytokines indicated in the figure using multiplex kits from Mesoscale discovery. Electrochemical luminescence was used as the detection system, and samples were analyzed according to the manufacturer’s protocol and read on a Sector 6000 plate reader. Results were calculated using the Mesoscale Workbench software package.

### Elastase degranulation assay

Neutrophils were resuspended PBS^+^ (11.10^6^/ml). 270 µl aliquots were transferred to 2-ml Eppendorf tubes and incubated at 37^°^C for 1 h. After 1 h, the cells were primed with cytochalasin B (5 µg/ml; Sigma-Aldrich) for 5 min, and then activated with 10 µM fMLP (Sigma-Aldrich) or vehicle for 10 min. Cells were pelleted, and the supernatant was transferred to fresh tubes. Samples were frozen and stored at −80°C until further analysis. Measurement of NE activity was performed using the EnChek activity assay (Molecular Probes), which is based on the cleavage of DQ-Elastin.

### Human macrophage differentiation and infection

An undifferentiated human iPS cell line was maintained on a monolayer of mitotically inactivated mouse embryonic feeder (MEF) cells in Advanced Dulbecco’s modified Eagles/F12 medium (DMEM/F12), supplemented with 20% knockout replacement serum (KSR), 2 mM l-Glutamine, 0.055 mM β-mercaptoethanol, and 8 ng/ml recombinant human FGF2 (R&D Systems) as described previously (van Wilgenburg et al., 2013). These cells were differentiated into macrophages following a previously published method (van Wilgenburg et al., 2013)). In brief, this protocol involves three key stages of differentiation: (1) formation of three germ layers containing embryoid bodies (EBs) from iPSCs on withdrawing FGF; (2) long-term production of myeloid precursor cells from EBs in presence of 25 ng/ml IL-3 and 50 ng/ml M-CSF (both R&D Systems); and (3) terminal differentiation and maturation of myeloid precursors into matured macrophagess in the presence of higher concentrations of M-CSF (100 ng/ml).

Matured macrophages were primed overnight with 20 ng/ml IFN-γ. On the day of infection, cells were washed with PBS and *S*. Typhimurium and were added to the media at indicated MOI 10 and incubated at 37°C for 1 h. After incubation, cells were washed three times and incubated for another hour with media containing 50 ng/ml Gentamicin to kill extracellular bacteria. Fresh media was added and incubated for an additional 4 h, and then cells were harvested and snap frozen for future analysis.

### Protein digestion and TMT labeling for human macrophages

Each cell pellet was lysed in 200 µl 0.1 M triethylammonium bicarbonate (TEAB), and then 0.1% SDS buffer with two rounds of pulsed probe sonication for 20 s, followed by 5 min boiling at 90°C. Cell debris was removed by centrifugation at 10,000 rpm for 10 min. Protein concentration was measured with Quick Start Bradford Protein Assay (Bio-Rad Laboratories) according to the manufacturer’s instructions. Aliquots containing 80 µg of total protein were prepared for trypsin digestion. Cysteine disulfide bonds were reduced by the addition of 2 µl 50 mM tris-2-carboxymethyl phosphine (TCEP), followed by 1 h incubation in heating block at 60°C. Cysteine residues were blocked by the addition of 1 µl 200 mM freshly prepared Iodoacetamide (IAA) solution and 30 min incubation at room temperature in dark. Trypsin (MS Grade; Thermo Fisher Scientific) solution was added at a final concentration 70 ng/µl to each sample for overnight digestion. After proteolysis, the peptide samples were diluted up to 100 µl with 0.1 M TEAB buffer. A 41-µl vol of anhydrous acetonitrile was added to each TMT 10-plex reagent (Thermo Fisher Scientific) vial and, after vortex mixing, the content of each TMT vial was transferred to each sample tube. Sample were labeled in biological replicates according to the following scheme: 127_N and 129_N, Unstimulated; 127_C and 129_C, INFG; 128_N and 130_N, *Salmonella*; and 128_C and 130_C, INFG and *Salmonella*. Labeling reaction was quenched with 8 µl 5% hydroxylamine for 15 min after 1 h incubation at room temperature. Samples were pooled, and the mixture was dried with speedvac concentrator and stored at −20°C until the high-pH Reverse Phase fractionation.

### Analysis for human macrophages

Offline peptide fractionation based on high pH Reverse Phase chromatography was performed using the XBridge C18 column (2.1 × 150 mm, 3.5 µm, 120 Å; Waters) on an Ultimate 3000 HPLC system (Dionex) equipped with autosampler. Mobile phase (A) was composed of 0.1% ammonium hydroxide and mobile phase (B) was composed of 100% acetonitrile and 0.1% ammonium hydroxide. The TMT-labeled peptide mixture was reconstituted in 100 µl mobile phase (A), centrifuged, and injected for fractionation. The multi-step gradient elution method at 0.2 ml/min was as follows: for 5 min isocratic at 5% (B), for 35 min gradient to 35% (B), gradient to 80% (B) in 5 min, isocratic for 5 min, and reequilibration to 5% (B). Signal was recorded at 280 nm and fractions were collected in a time-dependent manner every 1 min. The collected fractions were dried with SpeedVac concentrator and stored at −20°C until the LC-MS analysis.

LC-MS analysis was performed on the Ultimate 3000 UHPLC system (Dionex) coupled with the high-resolution LTQ Orbitrap Velos mass spectrometer (Thermo Fisher Scientific). Each peptide fraction was reconstituted in 40 µl 0.1% formic acid and a volume of 5 µl was loaded to the Acclaim PepMap 100, 100 µm × 2 cm C18, 5 µm, 100 Å trapping column with a user-modified injection method at 10 µl/min flow rate. The sample was then subjected to a multi-step gradient elution on the Acclaim PepMap RSLC (75 µm × 50 cm, 2 µm, 100 Å) C18 capillary column (Dionex) retrofitted to an electrospray emitter (New Objective) at 45°C. Mobile phase (A) was composed of 96% H_2_O, 4% DMSO, and 0.1% formic acid and mobile phase (B) was composed of 80% acetonitrile, 16% H_2_O, 4% DMSO, and 0.1% formic acid. The gradient separation method at flow rate 300 nl/min was as follows: for 95 min gradient to 45% B, for 5 min up to 95% B, for 8 min isocratic at 95% B, reequilibration to 5% B in 2 min, and for 10 min isocratic at 5% B.

The 10 most abundant multiply charged precursors within 380 −1,500 m/z were selected with FT mass resolution of 30,000 and isolated for HCD fragmentation with isolation width 1.2 Th. Normalized collision energy was set at 40, and the activation time was 0.1 ms for one microscan. Tandem mass spectra were acquired with FT resolution of 30,000, and targeted precursors were dynamically excluded for further isolation and activation for 40 s with 10 ppm mass tolerance. FT max ion time for full MS experiments was set at 200 ms, and FT MSn max ion time was set at 100 ms. The AGC target vales were 3 × 10^−6^ for full FTMS and 1 × 10^−5^ for MSn FTMS. The DMSO signal at m/z 401.922718 was used as a lock mass.

### Database search and protein quantification

The acquired mass spectra were submitted to SequestHT search engine implemented on the Proteome Discoverer 1.4 software for protein identification and quantification. The precursor mass tolerance was set at 30 ppm and the fragment ion mass tolerance was set at 0.02 D. TMT6plex at N terminus, and Carbamidomethyl at C terminus were defined as static modifications. Dynamic modifications included oxidation of M and Deamidation of N,Q. Peptide confidence was estimated with the Percolator node. Peptide FDR was set at 0.01, and validation was based on q-value and decoy database search. All spectra were searched against a UniProt fasta file containing 20,190 reviewed human entries. The Reporter Ion Quantifier node included a custom TMT 10plex Quantification Method with integration window tolerance 20 ppm and integration method the Most Confident Centroid.

### Mass spectrometry analysis of mouse neutrophils

Samples were resolved by SDS-PAGE, reduced, alkylated, and digested in-gel using trypsin. The resulting peptides were analyzed by LC-MSMS using either a Q Exactive (Thermo Fisher Scientific) coupled to an RSLC3000 UHPLC (Thermo Fisher Scientific) or an Orbitrap Fusion Tribrid (Thermo Fisher Scientific) coupled to an RSLC3000 UHPLC (Thermo Fisher Scientific). Data were acquired in a DDA fashion with quadrupole precursor selection and MS2 spectra in the Fusion acquired in the LTQ. Replicate runs were processed using Maxquant 1.5.2.8 searching a *Mus musculus* Uniprot database (downloaded 25/11/14, 73,415 entries) with protein N-terminal acetylation and methionine oxidation as variable modifications and carbamidomethyl cysteine as a fixed modification. Label-free quantitation was enabled, and the resulting data were processed in Perseus 1.5.1.6. Reverse and potential contaminant proteins were removed, and LFQ intensities were log_2_ transformed. Data were filtered so proteins required a minimum of three intensity values, and missing values were replaced from a normal distribution. Before imputation, the only protein with LFQ intensities in all control samples and no values in *Eros^−/−^* samples was BC017643. The resulting files were exported to *limma* for analysis.

### Online supplemental material

Fig. S1 shows identification of *bc017643/Eros* as a novel gene involved in susceptibility to *S.* Typhimurium. Fig. S2 shows that * Eros^−/−^* neutrophils can generate some reactive oxygen species when compared directly with those from gp91*phox^−/−^* and p22phox*^−/−^* mice, but BM-derived macrophages from *Eros^−/−^* mice have an almost absent response. Fig. S3 shows that * Eros^−/−^* mice are slightly less susceptible to *S.* Typhimurium infection in vivo than gp91*phox^−/−^* mice. Fig. S4 shows that Eros is the only differentially expressed gene when splenocytes from control and *Eros^−/−^* mice are compared by microarray. Fig. S5 shows that densitometry plots for data shown in Fig. 7 (B–D) and only modest rescue of gp91 with inhibitors of protein degradation in *Eros^−/−^* cells and constructs used for co-localization and pull-down experiments.

## Supplementary Material

Supplemental Materials (PDF)

## References

[bib1] Ayadi A., Birling M.C., Bottomley J., Bussell J., Fuchs H., Fray M., Gailus-Durner V., Greenaway S., Houghton R., Karp N., 2012 Mouse large-scale phenotyping initiatives: overview of the European Mouse Disease Clinic (EUMODIC) and of the Wellcome Trust Sanger Institute Mouse Genetics Project. Mamm. Genome. 23:600–610. 10.1007/s00335-012-9418-y 22961258 PMC3463797

[bib2] Babior B.M. 1984 The respiratory burst of phagocytes. J. Clin. Invest. 73:599–601. 10.1172/JCI111249 6323522 PMC425058

[bib3] Berendes H., Bridges R.A., and Good R.A. 1957 A fatal granulomatosus of childhood: the clinical study of a new syndrome. Minn. Med. 40:309–312. 13430573

[bib4] Boudreau E., Takahashi Y., Lemieux C., Turmel M., and Rochaix J.D. 1997 The chloroplast ycf3 and ycf4 open reading frames of Chlamydomonas reinhardtii are required for the accumulation of the photosystem I complex. EMBO J. 16:6095–6104. 10.1093/emboj/16.20.6095 9321389 PMC1326293

[bib5] Broz P., Ruby T., Belhocine K., Bouley D.M., Kayagaki N., Dixit V.M., and Monack D.M. 2012 Caspase-11 increases susceptibility to Salmonella infection in the absence of caspase-1. Nature. 490:288–291. 10.1038/nature11419 22895188 PMC3470772

[bib6] Burton N.A., Schürmann N., Casse O., Steeb A.K., Claudi B., Zankl J., Schmidt A., and Bumann D. 2014 Disparate impact of oxidative host defenses determines the fate of Salmonella during systemic infection in mice. Cell Host Microbe. 15:72–83. 10.1016/j.chom.2013.12.006 24439899

[bib7] Cale C.M., Morton L., and Goldblatt D. 2007 Cutaneous and other lupus-like symptoms in carriers of X-linked chronic granulomatous disease: incidence and autoimmune serology. Clin. Exp. Immunol. 148:79–84. 10.1111/j.1365-2249.2007.03321.x 17286762 PMC1868856

[bib8] Campbell A.M., Kashgarian M., and Shlomchik M.J. 2012 NADPH oxidase inhibits the pathogenesis of systemic lupus erythematosus. Sci. Transl. Med. 4:157ra141 10.1126/scitranslmed.3004801 PMC3704198 23100627

[bib9] Chatfield S.N., Strahan K., Pickard D., Charles I.G., Hormaeche C.E., and Dougan G. 1992 Evaluation of *Salmonella* Typhimurium strains harbouring defined mutations in htrA and aroA in the murine salmonellosis model. Microb. Pathog. 12:145–151. 10.1016/0882-4010(92)90117-7 1584006

[bib10] Chen F., Pandey D., Chadli A., Catravas J.D., Chen T., and Fulton D.J. 2011 Hsp90 regulates NADPH oxidase activity and is necessary for superoxide but not hydrogen peroxide production. Antioxid. Redox Signal. 14:2107–2119. 10.1089/ars.2010.3669 21194376 PMC3085945

[bib11] Chen F., Yu Y., Qian J., Wang Y., Cheng B., Dimitropoulou C., Patel V., Chadli A., Rudic R.D., Stepp D.W., 2012 Opposing actions of heat shock protein 90 and 70 regulate nicotinamide adenine dinucleotide phosphate oxidase stability and reactive oxygen species production. Arterioscler. Thromb. Vasc. Biol. 32:2989–2999. 10.1161/ATVBAHA.112.300361 23023377 PMC3499642

[bib12] Conlan J.W. 1997 Critical roles of neutrophils in host defense against experimental systemic infections of mice by *Listeria monocytogenes*, *Salmonella* Typhimurium, and *Yersinia enterocolitica*. Infect. Immun. 65:630–635. 9009323 10.1128/iai.65.2.630-635.1997 PMC176106

[bib13] DeLeo F.R., Burritt J.B., Yu L., Jesaitis A.J., Dinauer M.C., and Nauseef W.M. 2000 Processing and maturation of flavocytochrome b558 include incorporation of heme as a prerequisite for heterodimer assembly. J. Biol. Chem. 275:13986–13993. 10.1074/jbc.275.18.13986 10788525

[bib14] Dill B.D., Gierlinski M., Härtlova A., Arandilla A.G., Guo M., Clarke R.G., and Trost M. 2015 Quantitative proteome analysis of temporally resolved phagosomes following uptake via key phagocytic receptors. Mol. Cell. Proteomics. 14:1334–1349. 10.1074/mcp.M114.044594 25755298 PMC4424403

[bib15] Dinauer M.C., Deck M.B., and Unanue E.R. 1997 Mice lacking reduced nicotinamide adenine dinucleotide phosphate oxidase activity show increased susceptibility to early infection with Listeria monocytogenes. J. Immunol. 158:5581–5583. 9190903

[bib16] Dougan G., John V., Palmer S., and Mastroeni P. 2011 Immunity to salmonellosis. Immunol. Rev. 240:196–210. 10.1111/j.1600-065X.2010.00999.x 21349095

[bib17] Fattouh R., Guo C.H., Lam G.Y., Gareau M.G., Ngan B.Y., Glogauer M., Muise A.M., and Brumell J.H. 2013 Rac2-deficiency leads to exacerbated and protracted colitis in response to Citrobacter rodentium infection. PLoS One. 8:e61629 10.1371/journal.pone.0061629 23613889 PMC3628927

[bib18] Finn R.D., Clements J., and Eddy S.R. 2011 HMMER web server: interactive sequence similarity searching. Nucleic Acids Res. 39(suppl):W29-37 10.1093/nar/gkr367 21593126 PMC3125773

[bib19] Gilchrist J.J., MacLennan C.A., and Hill A.V. 2015 Genetic susceptibility to invasive Salmonella disease. Nat. Rev. Immunol. 15:452–463. 10.1038/nri3858 26109132

[bib20] Jacob C.O., Eisenstein M., Dinauer M.C., Ming W., Liu Q., John S., Quismorio F.P. Jr., Reiff A., Myones B.L., Kaufman K.M., 2012 Lupus-associated causal mutation in neutrophil cytosolic factor 2 (NCF2) brings unique insights to the structure and function of NADPH oxidase. Proc. Natl. Acad. Sci. USA. 109:E59–E67. 10.1073/pnas.1113251108 22203994 PMC3258621

[bib21] Joshi S., Singh A.R., Zulcic M., Bao L., Messer K., Ideker T., Dutkowski J., and Durden D.L. 2014 Rac2 controls tumor growth, metastasis and M1-M2 macrophage differentiation in vivo. PLoS One. 9:e95893 10.1371/journal.pone.0095893 24770346 PMC4000195

[bib22] Käll L., Krogh A., and Sonnhammer E.L. 2004 A combined transmembrane topology and signal peptide prediction method. J. Mol. Biol. 338:1027–1036. 10.1016/j.jmb.2004.03.016 15111065

[bib23] Kelkka T., Pizzolla A., Laurila J.P., Friman T., Gustafsson R., Källberg E., Olsson O., Leanderson T., Rubin K., Salmi M., 2013 Mice lacking NCF1 exhibit reduced growth of implanted melanoma and carcinoma tumors. PLoS One. 8:e84148 10.1371/journal.pone.0084148 24358335 PMC3865299

[bib24] Kim C., and Dinauer M.C. 2006 Impaired NADPH oxidase activity in Rac2-deficient murine neutrophils does not result from defective translocation of p47phox and p67phox and can be rescued by exogenous arachidonic acid. J. Leukoc. Biol. 79:223–234. 10.1189/jlb.0705371 16275890

[bib25] Kim M.S., Pinto S.M., Getnet D., Nirujogi R.S., Manda S.S., Chaerkady R., Madugundu A.K., Kelkar D.S., Isserlin R., Jain S., 2014 A draft map of the human proteome. Nature. 509:575–581. 10.1038/nature13302 24870542 PMC4403737

[bib26] Klebanoff S.J. 1967 Iodination of bacteria: a bactericidal mechanism. J. Exp. Med. 126:1063–1078. 10.1084/jem.126.6.1063 4964565 PMC2138423

[bib27] Klebanoff S.J., Clem W.H., and Luebke R.G. 1966 The peroxidase-thiocyanate-hydrogen peroxide antimicrobial system. Biochim. Biophys. Acta. 117:63–72. 10.1016/0304-4165(66)90152-8 4380562

[bib28] Krech K., Ruf S., Masduki F.F., Thiele W., Bednarczyk D., Albus C.A., Tiller N., Hasse C., Schöttler M.A., and Bock R. 2012 The plastid genome-encoded Ycf4 protein functions as a nonessential assembly factor for photosystem I in higher plants. Plant Physiol. 159:579–591. 10.1104/pp.112.196642 22517411 PMC3375926

[bib29] Mastroeni P., Vazquez-Torres A., Fang F.C., Xu Y., Khan S., Hormaeche C.E., and Dougan G. 2000 Antimicrobial actions of the NADPH phagocyte oxidase and inducible nitric oxide synthase in experimental salmonellosis. II. Effects on microbial proliferation and host survival in vivo. J. Exp. Med. 192:237–248. 10.1084/jem.192.2.237 10899910 PMC2193252

[bib30] Nakamura N., Lill J.R., Phung Q., Jiang Z., Bakalarski C., de Mazière A., Klumperman J., Schlatter M., Delamarre L., and Mellman I. 2014 Endosomes are specialized platforms for bacterial sensing and NOD2 signalling. Nature. 509:240–244. 10.1038/nature13133 24695226

[bib31] Nakano Y., Onozuka K., Terada Y., Shinomiya H., and Nakano M. 1990 Protective effect of recombinant tumor necrosis factor-alpha in murine salmonellosis. J. Immunol. 144:1935–1941. 2106556

[bib32] Nakano Y., Longo-Guess C.M., Bergstrom D.E., Nauseef W.M., Jones S.M., and Bánfi B. 2008 Mutation of the Cyba gene encoding p22phox causes vestibular and immune defects in mice. J. Clin. Invest. 118:1176–1185. 18292807 10.1172/JCI33835 PMC2248803

[bib33] Noubade R., Wong K., Ota N., Rutz S., Eidenschenk C., Valdez P.A., Ding J., Peng I., Sebrell A., Caplazi P., 2014 NRROS negatively regulates reactive oxygen species during host defence and autoimmunity. Nature. 509:235–239. 10.1038/nature13152 24739962

[bib34] Okada F., Kobayashi M., Tanaka H., Kobayashi T., Tazawa H., Iuchi Y., Onuma K., Hosokawa M., Dinauer M.C., and Hunt N.H. 2006 The role of nicotinamide adenine dinucleotide phosphate oxidase-derived reactive oxygen species in the acquisition of metastatic ability of tumor cells. Am. J. Pathol. 169:294–302. 10.2353/ajpath.2006.060073 16816381 PMC1698756

[bib35] Pollock J.D., Williams D.A., Gifford M.A., Li L.L., Du X., Fisherman J., Orkin S.H., Doerschuk C.M., and Dinauer M.C. 1995 Mouse model of X-linked chronic granulomatous disease, an inherited defect in phagocyte superoxide production. Nat. Genet. 9:202–209. 10.1038/ng0295-202 7719350

[bib36] Rae J., Noack D., Heyworth P.G., Ellis B.A., Curnutte J.T., and Cross A.R. 2000 Molecular analysis of 9 new families with chronic granulomatous disease caused by mutations in CYBA, the gene encoding p22(phox). Blood. 96:1106–1112. 10910929

[bib37] Remijsen Q., Vanden Berghe T., Wirawan E., Asselbergh B., Parthoens E., De Rycke R., Noppen S., Delforge M., Willems J., and Vandenabeele P. 2011 Neutrophil extracellular trap cell death requires both autophagy and superoxide generation. Cell Res. 21:290–304. 10.1038/cr.2010.150 21060338 PMC3193439

[bib38] Richter-Dahlfors A., Buchan A.M., and Finlay B.B. 1997 Murine salmonellosis studied by confocal microscopy: *Salmonella* Typhimurium resides intracellularly inside macrophages and exerts a cytotoxic effect on phagocytes in vivo. J. Exp. Med. 186:569–580. 10.1084/jem.186.4.569 9254655 PMC2199036

[bib39] Roberts A.W., Kim C., Zhen L., Lowe J.B., Kapur R., Petryniak B., Spaetti A., Pollock J.D., Borneo J.B., Bradford G.B., 1999 Deficiency of the hematopoietic cell-specific Rho family GTPase Rac2 is characterized by abnormalities in neutrophil function and host defense. Immunity. 10:183–196. 10.1016/S1074-7613(00)80019-9 10072071

[bib40] Rydström A., and Wick M.J. 2010 Salmonella inhibits monocyte differentiation into CD11c hi MHC-II hi cells in a MyD88-dependent fashion. J. Leukoc. Biol. 87:823–832. 10.1189/jlb.0909615 20124491

[bib41] Segal A.W. 1987 Absence of both cytochrome b-245 subunits from neutrophils in X-linked chronic granulomatous disease. Nature. 326:88–91. 10.1038/326088a0 3821877

[bib42] Segal A.W. 2005 How neutrophils kill microbes. Annu. Rev. Immunol. 23:197–223. 10.1146/annurev.immunol.23.021704.115653 15771570 PMC2092448

[bib43] Singel K.L., and Segal B.H. 2016 NOX2-dependent regulation of inflammation. Clin. Sci. 130:479–490. 10.1042/CS20150660 26888560 PMC5513728

[bib44] Skarnes W.C., Rosen B., West A.P., Koutsourakis M., Bushell W., Iyer V., Mujica A.O., Thomas M., Harrow J., Cox T., 2011 A conditional knockout resource for the genome-wide study of mouse gene function. Nature. 474:337–342. 10.1038/nature10163 21677750 PMC3572410

[bib45] Tam M.A., Rydström A., Sundquist M., and Wick M.J. 2008 Early cellular responses to Salmonella infection: dendritic cells, monocytes, and more. Immunol. Rev. 225:140–162. 10.1111/j.1600-065X.2008.00679.x 18837781

[bib46] Teahan C., Rowe P., Parker P., Totty N., and Segal A.W. 1987 The X-linked chronic granulomatous disease gene codes for the beta-chain of cytochrome b-245. Nature. 327:720–721. 10.1038/327720a0 3600769

[bib47] Trost M., English L., Lemieux S., Courcelles M., Desjardins M., and Thibault P. 2009 The phagosomal proteome in interferon-gamma-activated macrophages. Immunity. 30:143–154. 10.1016/j.immuni.2008.11.006 19144319

[bib48] van den Berg J.M., van Koppen E., Ahlin A., Belohradsky B.H., Bernatowska E., Corbeel L., Español T., Fischer A., Kurenko-Deptuch M., Mouy R., 2009 Chronic granulomatous disease: the European experience. PLoS One. 4:e5234 10.1371/journal.pone.0005234 19381301 PMC2668749

[bib49] van Wilgenburg B., Browne C., Vowles J., and Cowley S.A. 2013 PLoS One. 8:e71098 10.1371/journal.pone.0071098 23951090 PMC3741356

[bib50] Vazquez-Torres A., Jones-Carson J., Mastroeni P., Ischiropoulos H., and Fang F.C. 2000 a Antimicrobial actions of the NADPH phagocyte oxidase and inducible nitric oxide synthase in experimental salmonellosis. I. Effects on microbial killing by activated peritoneal macrophages in vitro. J. Exp. Med. 192:227–236. 10.1084/jem.192.2.227 10899909 PMC2193262

[bib51] Vazquez-Torres A., Xu Y., Jones-Carson J., Holden D.W., Lucia S.M., Dinauer M.C., Mastroeni P., and Fang F.C. 2000 b Salmonella pathogenicity island 2-dependent evasion of the phagocyte NADPH oxidase. Science. 287:1655–1658. 10.1126/science.287.5458.1655 10698741

[bib52] Vazquez-Torres A., Vallance B.A., Bergman M.A., Finlay B.B., Cookson B.T., Jones-Carson J., and Fang F.C. 2004 Toll-like receptor 4 dependence of innate and adaptive immunity to Salmonella: importance of the Kupffer cell network. J. Immunol. 172:6202–6208. 10.4049/jimmunol.172.10.6202 15128808

[bib53] Vidal S., Tremblay M.L., Govoni G., Gauthier S., Sebastiani G., Malo D., Skamene E., Olivier M., Jothy S., and Gros P. 1995 The Ity/Lsh/Bcg locus: natural resistance to infection with intracellular parasites is abrogated by disruption of the Nramp1 gene. J. Exp. Med. 182:655–666. 10.1084/jem.182.3.655 7650477 PMC2192162

[bib54] White J.K., Gerdin A.K., Karp N.A., Ryder E., Buljan M., Bussell J.N., Salisbury J., Clare S., Ingham N.J., Podrini C., Sanger Institute Mouse Genetics Project 2013 Genome-wide generation and systematic phenotyping of knockout mice reveals new roles for many genes. Cell. 154:452–464. 10.1016/j.cell.2013.06.022 23870131 PMC3717207

[bib55] Yamauchi A., Kim C., Li S., Marchal C.C., Towe J., Atkinson S.J., and Dinauer M.C. 2004 Rac2-deficient murine macrophages have selective defects in superoxide production and phagocytosis of opsonized particles. J. Immunol. 173:5971–5979. 10.4049/jimmunol.173.10.5971 15528331

