# Supplementary material for: Eros is a novel transmembrane protein that controls the phagocyte respiratory burst and is essential for innate immunity
Source: J Exp Med. 2017 Apr 3;214(4):1111–28. doi: 10.1084/jem.20161382 (PMC5379978; doi:10.1084/jem.20161382)
Supplement: Supplemental Materials (PDF) [file JEM_20161382_sm.pdf]

The Journal of Experimental Medicine

SUPPLEMENTAL MATERIAL

Thomas et al., https://doi.org/10.1084/jem.20161382

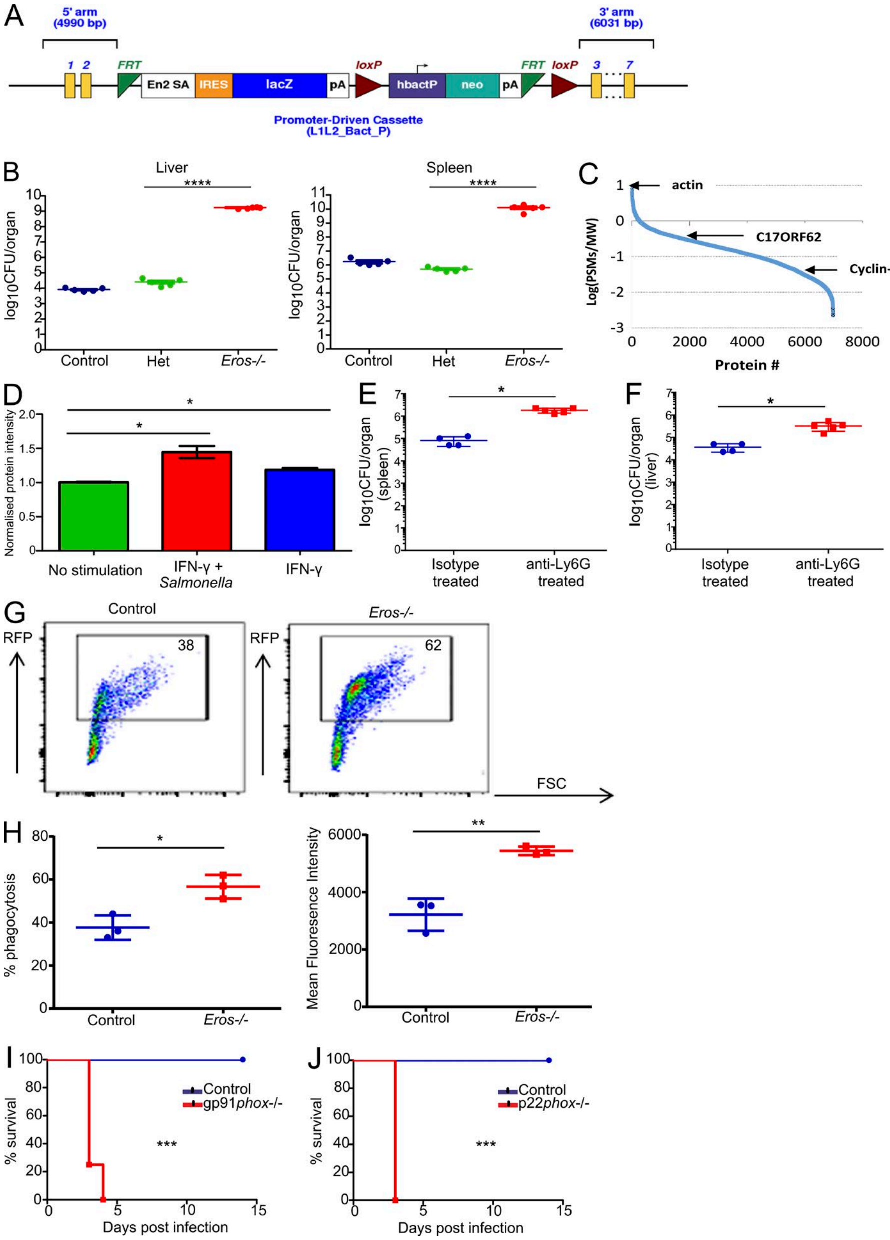

Figure S1. **Identification of bc017643/Eros as a novel gene involved in susceptibility to *S.* Typhimurium: the human ortholog of *Eros* is expressed in macrophages and up-regulated by *Salmonella* and IFN-γ.** (A) Schematic of the vector used to target *Eros* in mouse embryonic stem cells. (B) Bacterial burden 4 d after infection with *S.* Typhimurium M525 in the indicated tissues in control (five mice), heterozygote (het; five mice), and *Eros*$^{-/-}$ mice (five mice). Data are representative of two independent experiments. (C and D) Abundance of *C17ORF62* measured by mass spectrometry compared with other detected proteins in human iPS-derived macrophages at baseline (C) and after stimulation with IFN-γ or a combination of *Salmonella* and IFN-γ (D). (E and F) Bacterial burden at 3 d after infection with *S.* Typhimurium M525 in spleen (E) and liver (F) in mice treated with an anti–neutrophil-depleting antibody (1A8; five mice) or an isotype control antibody (five mice). Data are representative of two separate experiments. (G) Representative plots of purified BM-derived neutrophils from control or *Eros*$^{-/-}$ mice 3 h after infection with a constitutive RFP-expressing strain of *S.* Typhimurium SL1344. (H) Percentage of neutrophils infected with bacteria and mean fluoresence intensity of infected cells at 3 h after infection. Each data point represents a biological replicate, and data were analyzed by unpaired Student's *t* test. Data are representative of two separate experiments. (G) Survival of gp91*phox*$^{-/-}$ and p22*phox*$^{-/-}$ mice after infection with *S.* Typhimurium M525. Error bars represent the SEM. *, P < 0.05; **, P < 0.01; ***, P < 0.001; ****, P < 0.0001. Data in B, C, E, and F were analyzed by Mann-Whitney test and data in D and F were analyzed by unpaired Student's *t* test.

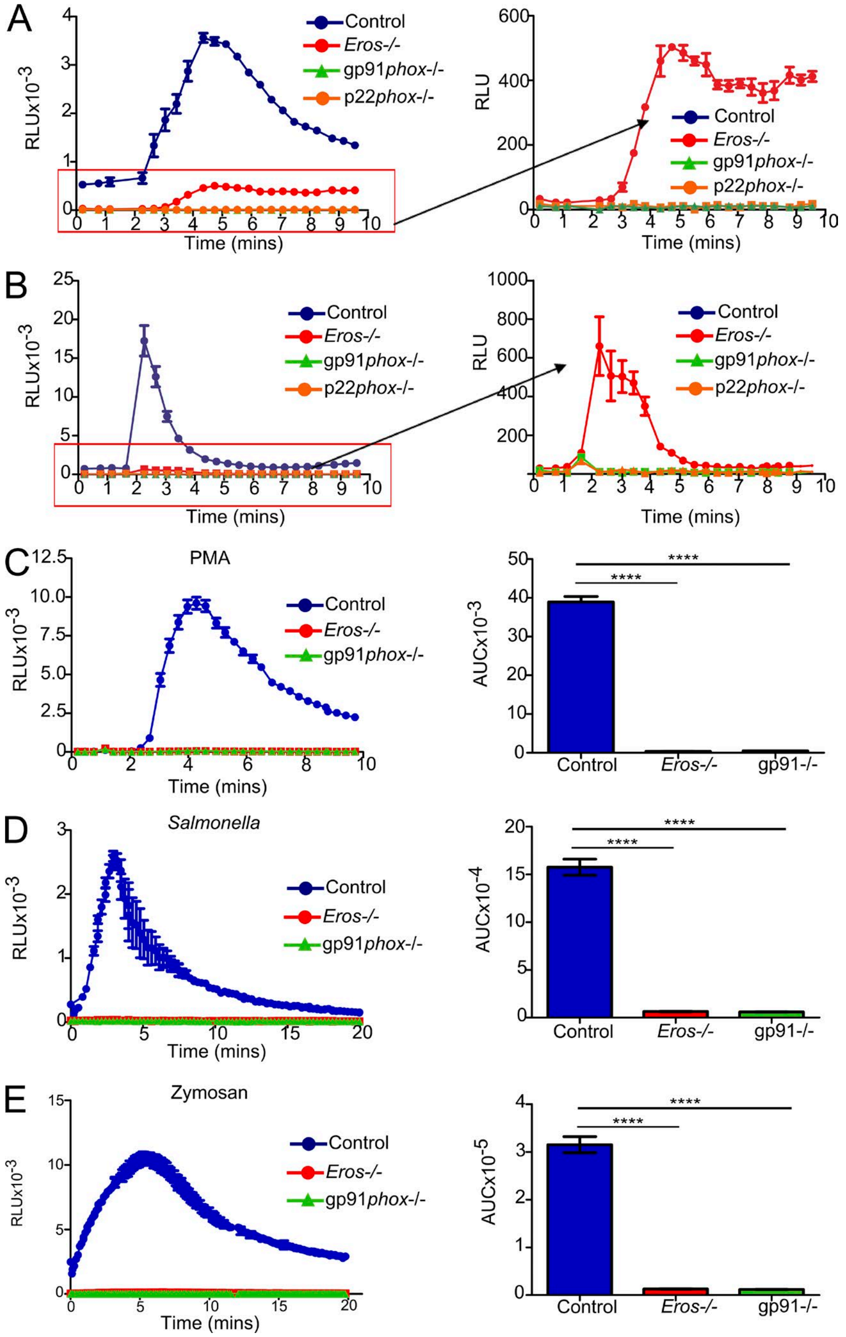

Figure S2. ***Eros*$^{-/-}$ neutrophils can generate some reactive oxygen species when compared directly with those from gp91$^{-/-}$ and p22$^{-/-}$ mice, but BM-derived macrophages from *Eros*$^{-/-}$ mice have an almost absent response.** (A and B) ROS production measured in relative light units (RLU) by purified BM neutrophils from control, *Eros*$^{-/-}$, gp91$^{-/-}$ (*Cybb*$^{-/-}$), or p22$^{-/-}$ (*Cyba*$^{-/-}$) mice in response to PMA (A) or fMLP (B). (right) The same graphs with control samples removed to highlight the difference between Eros$^{-/-}$ and gp91$^{-/-}$ or p22$^{-/-}$. Data are representative of three technical replicates. ROS response of BM-derived macrophages from control, *Eros*$^{-/-}$, and gp91$^{-/-}$ mice in response to PMA (C), *S.* Typhimurium M525 (D), and Zymosan (E). Data are representative of three technical replicates. Area under the curve (AUC) was calculated for each sample and analyzed by unpaired Student's *t* test. Error bars represent the SEM. ****, $P < 0.0001$. Data are representative of three independent experiments.

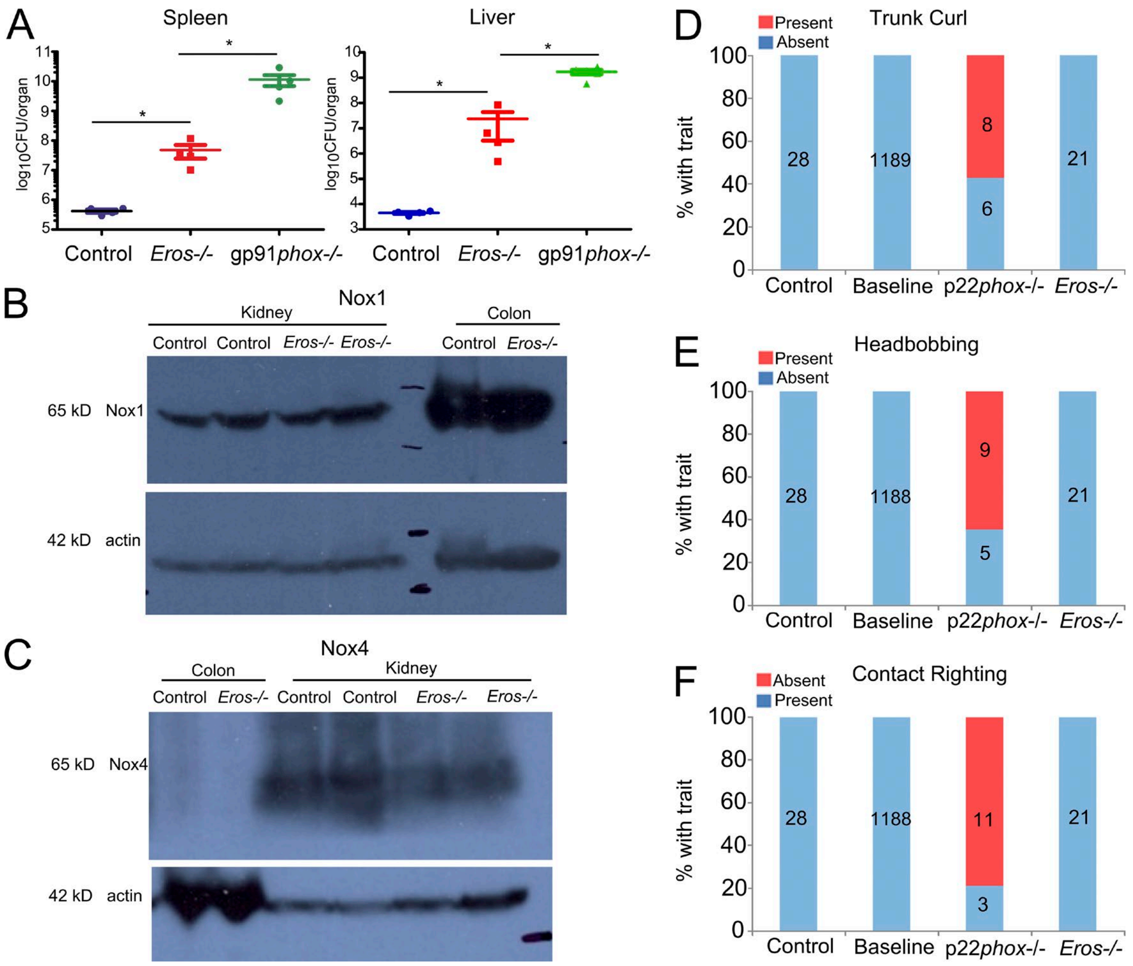

Figure S3. ***Eros***$^{-/-}$ **mice are slightly less susceptible to *S.* Typhimurium infection in vivo than gp91phox**$^{-/-}$ **mice.** Nox1 and Nox4 are equivalently expressed in control and Eros-deficient cells and Nox3 is functionally unaffected in *Eros*$^{-/-}$ cells. (A) Bacterial burden expressed as CFU per organ in spleen (A) and liver (B) at 72 h after infection with *S.* Typhimurium M525 and bacterial burden at 72 h after infection. Error bars represent the SEM. ****, $P < 0.0001$. Four mice were used in each group. Data were analyzed by Mann-Whitney test and are representative of three independent experiments. Nox1 expression (B) and Nox 4 in lysates of kidney and colon (C), as indicated from control and *Eros*$^{-/-}$ cells by Western blot. Actin expression is shown as a loading control. Data are representative of two independent experiments and each band represents a biological replicate. (D–F) Vestibular responses in control, *Eros*$^{-/-}$ and p22*phox*$^{-/-}$ mice presence of trunk curl (D), presence of head-bobbing/circling (E), and presence of an intact contact-righting reflex (F). Numbers on the graphs represent the numbers of mice in each group. "Baseline" represents data from all mice assessed as part of the Knockout Mouse Project (KOMP).

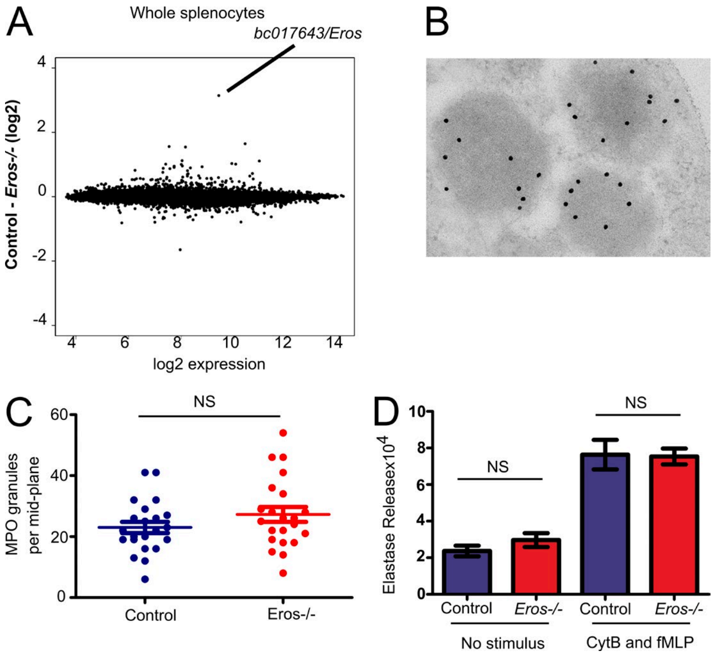

Figure S4. **Eros is the only differentially expressed gene when splenocytes from control and *Eros*$^{-/-}$ mice are compared by microarray.** (A) mRNA microarray analysis of splenocytes from control (seven individuals) and *Eros*$^{-/-}$ (eight individuals) mice showing *bc017643* (*Eros*) as the only statistically significant differentially expressed gene. (B) Number of myeloperoxidase (MPO) molecules in primary granules of Control or *Eros*$^{-/-}$ mice. Myeloperoxidase molecules are indicated with an arrow. (C) Elastase production by BM neutrophils from Control or *Eros*$^{-/-}$ mice after priming with cytocholasin B and stimulation with fMLP. Error bars represent the SEM. NS, nonsignificant. Data in C were analyzed by unpaired Student's *t* test.

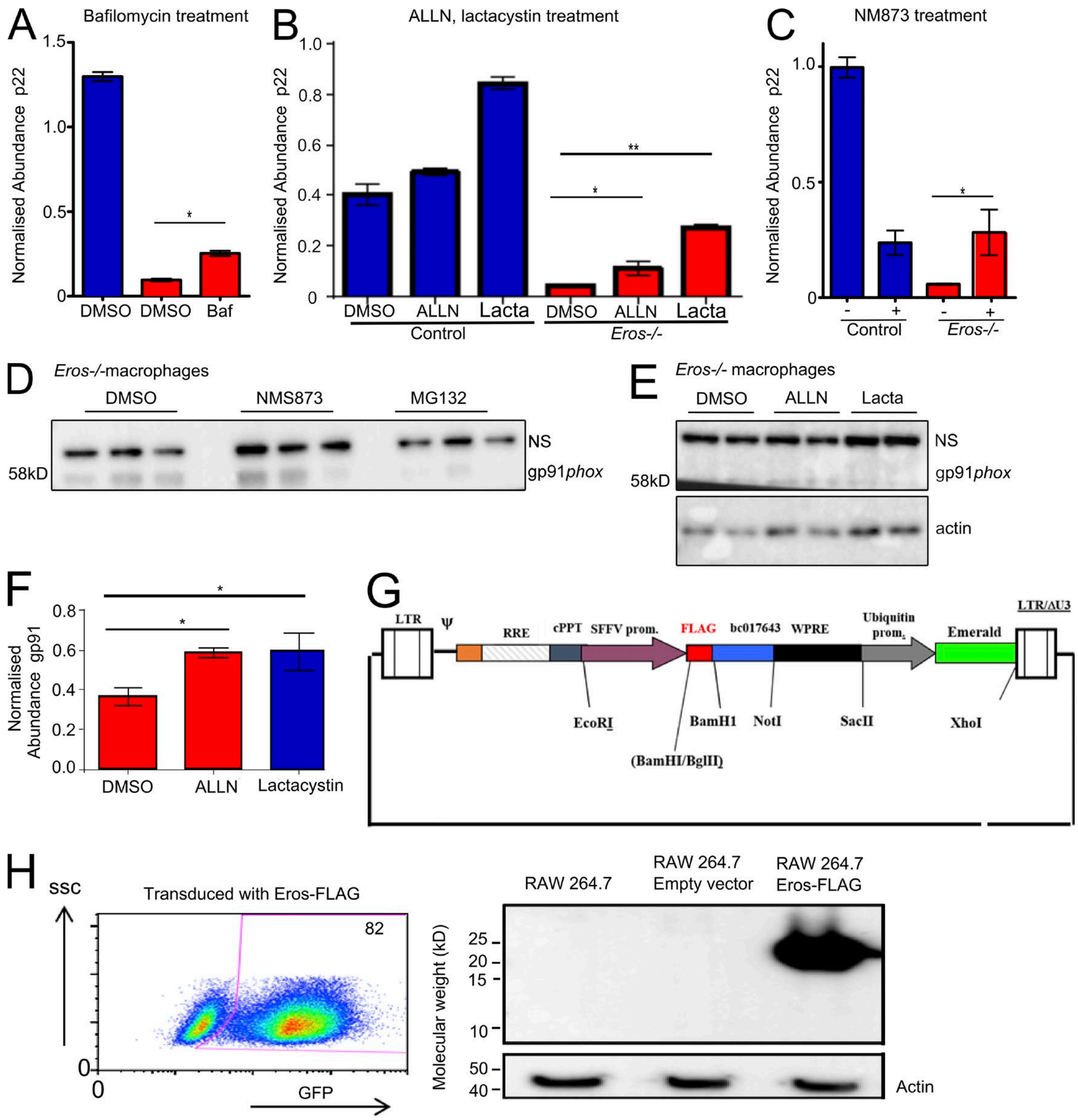

Figure S5. **Densitometry plots for data shown in Fig. 7 (B–D), showing only modest rescue of gp91 with inhibitors of protein degradation in *Eros*$^{-/-}$ cells and constructs used for co-localization and pull-down experiments.** (A–C) Densitometry plots for p22*phox* expression in macrophages in the presence of the indicated inhibitors of protein degradation. Three technical replicates were used in each case. (D and E) macrophage gp91*phox* expression in the presence of the indicated inhibitors of protein degradation. Data were analyzed by unpaired Student's *t* test and are representative of three independent experiments. Error bars represent the SEM. *, P < 0.05; **, P < 0.01. Data in B, C, and E were analyzed by unpaired Student's *t* test. (G) Schematic of the lentiviral vector used for transduction of RAW264.7 cells (H) transduction of RAW264.7 cells with *Eros*-FLAG and Western blot demonstrating overexpression of *Eros*-FLAG compared with cells transduced with empty vector.
